# Monogenic autoinflammatory diseases in children: single center experience with clinical, genetic, and imaging review

**DOI:** 10.1186/s13244-020-00889-0

**Published:** 2020-07-31

**Authors:** Alaa N. Alsharief, Ronald M. Laxer, Qiuyan Wang, Jennifer Stimec, Carina Man, Paul Babyn, Andrea S. Doria

**Affiliations:** 1 Department of Diagnostic Imaging, The Hospital for Sick Children, University of Toronto, Toronto, Canada; 2 Department of Paediatric, Division of Rheumatology, The Hospital for Sick Children, University of Toronto, Toronto, ON Canada; 3 York Radiology Consultants, Toronto, ON Canada; 4 Department of Medical Imaging, Royal University Hospital, Saskatoon, SK Canada; 5 Medical Imaging Department, Ministry of National Guard Health Affairs, King Saud bin Abdulaziz University for Health Sciences, King Abdulaziz Medical City - Western Region, Old Makkah Road Kilo 24 1 Western, P.O. Box 9515, Jeddah, 21423 Saudi Arabia

**Keywords:** Autoinflammatory diseases, Rheumatology, Radiology, Fever, Children

## Abstract

**Purpose:**

1. To review the contemporary literature and present a list of the imaging findings for patients with autoinflammatory diseases from our hospital. All these patients are found to have a genetic mutation that is responsible for their disease.

2. To present follow-up imaging findings, when available, and correlate those with symptoms and type of treatment administered in approximately 40 patients with autoinflammatory diseases of a single tertiary pediatric health care center including familial Mediterranean fever, Cryopyrin-associated autoinflammatory syndrome, PAPA (pyogenic arthritis, pyoderma gangrenousum, and acne) syndrome, and more. These findings are related to disease progression, treatment response, or treatment-induced changes.

**Conclusion:**

Autoinflammatory diseases are relatively rare entities that can affect any system of the body. Given the many nonspecific imaging features, awareness of these diseases and good communication with clinicians aid in reaching an accurate diagnosis.

## Key points

- Autoinflammatory diseases are systemic diseases that may affect any body part.
- Imaging findings are not specific in patients with these disease.
- Awareness of the clinical and imaging findings may assist in differential diagnosis.
- Early diagnosis and treatment decrease morbidity and mortality.

## Introduction

Inflammation is the complex biological response triggered as a protective attempt by the organism to remove injurious stimuli and to initiate healing. This response is normally closely regulated by the body, but in some cases there is an excessive innate immune response to known or unknown triggers, that can lead to episodic systemic and organ-specific inflammation.

Autoinflammatory diseases are considered rare and they encompass a wide variety of diseases and syndromes that are defined as self-directed inflammation, whereby local factors at sites predispose to disease leading to activation of innate immune cells, including macrophages and neutrophils, with resultant target tissue damage. These diseases are clinically characterized by chronic or recurrent episodes of inflammation without evidence of the typical features of the autoimmune diseases such as high titer autoantibodies or autoreactive lymphocytes [1].

The differential diagnoses in patients with autoinflammatory diseases include infections, malignancy, autoimmune diseases, and histiocytic disorders. Patients are usually investigated for one or more of these diseases before reaching the correct diagnosis.

**Fig. 1:**
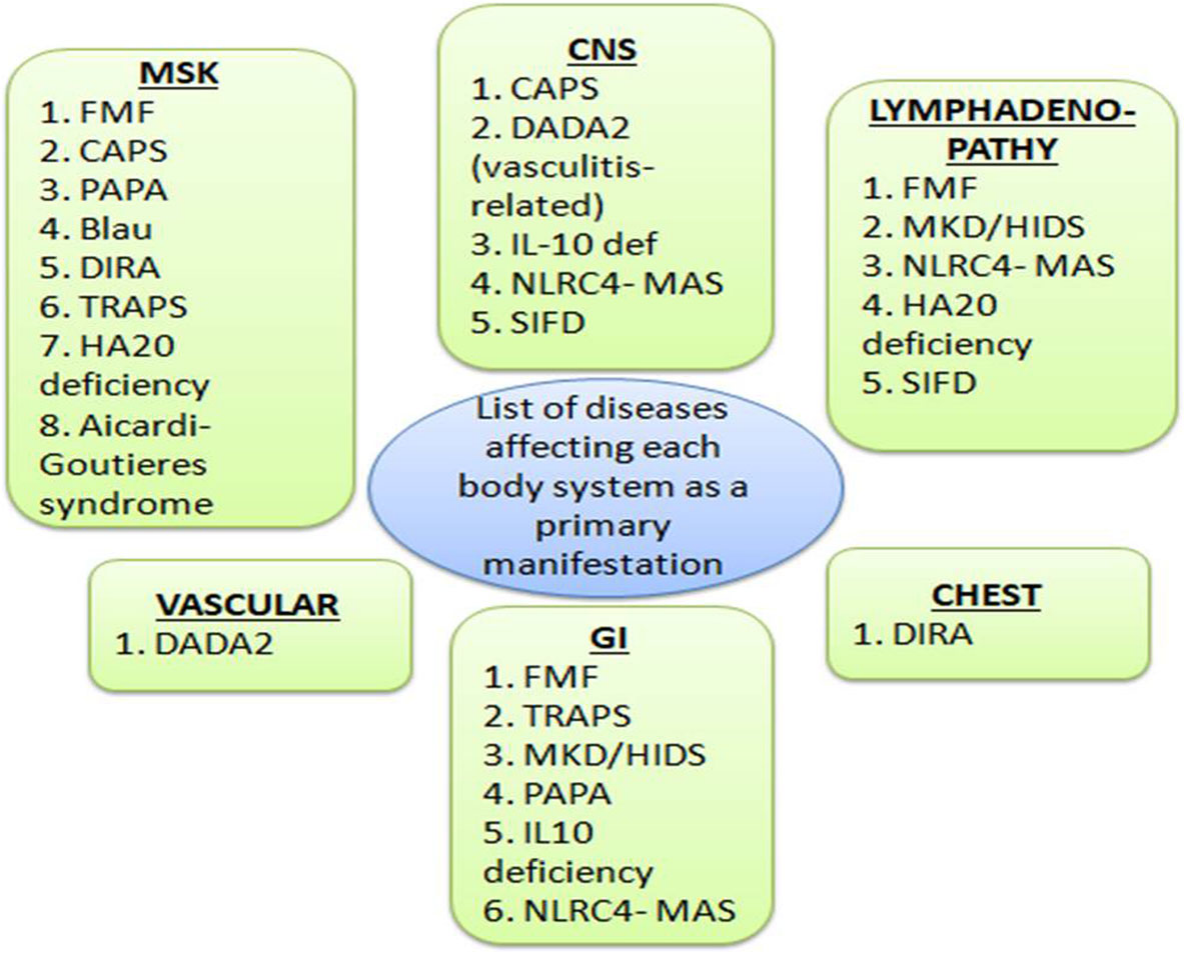
List of autoinflammatory diseases discussed in the paper of patients of our cohort according to the body system involved as a primary disease-related manifestation and specific locations in these body systems

Understanding the pathophysiologic mechanisms and imaging characteristics of autoinflammatory disorders in relation to key components of the inflammatory cascade is essential not only for diagnosis but also for the evaluation of new therapeutic and imaging strategies for inflammation in clinical trials and for management of inflammatory diseases. Many autoinflammatory diseases result from gene mutations that are directly or indirectly involved in the regulation of the interleukin1 (IL-1) cytokine signaling pathway. IL-1 is the prototype of a proinflammatory “alarm” cytokine that coordinates responses to endogenous and exogenous danger to the organism. IL-1α and IL-1β can bind to the IL-1R type1 receptor, which recruits the accessory receptor (IL-1RAcP). This receptor complex forms a signaling unit, which is part of the more complex signaling process in the inflammation cascade. IL-1 receptor antagonists such as anakinra can prevent this signal transduction and therefore effectively treat IL-1-induced autoinflammatory diseases [2]. Table 1 presents classification of the autoinflammatory diseases—of our patients—according to the disease mechanism. Figure 1 lists the diseases according to the body system involved on imaging as a primary disease-related manifestation.

**Table 1:** Classification of the autoinflammatory diseases of the patients of our cohort according to the immune mechanism responsible for mediating the disease process (with modifications from Manthiram et al. 2017)

I. Autoinflammatory diseases mediated by activated inflammosomes and IL1β production	
1. FMF 2. CAS 3. MKD/HIDS 4. PAPA 5. NLRC4-MAS 6. DIRA	
II. Autoinflammatory diseases mediated by the NF-κB pathway	
1. Blau syndrome 2. HA20 deficiency	
III. Autoinflammatory diseases mediated by the TNF pathway	
1. TRAPS 2. IL-10 Deficiency	
IV. Autoinflammatory disease mediated by the type I interferon pathway	
1. AGS	
V. Autoinflammatory diseases with unique not well established mechanism (to be included in the coming classifications)	
1. DADA2 2. SIFD	

*FMF* familial Mediterranean fever; *TRAPS* tumor necrosis factor receptor-associated periodic syndrome; *MKD* Mevalonate kinase deficiency; *HIDS* hyperimmunoglobulinemia D syndrome; *NOMID* neonatal onset multisystem inflammatory disease; *MWS* Muckle-Wells syndrome; *PAPA* pyogenic arthritis, pyoderma gangrenosum and acne; *DIRA* deficiency of interleukin-1 receptor antagonist; *DADA2* deficiency of adenosine deaminase type 2; *IL10 def* interleukin 10 deficiency; *MAS* macrophage activation syndrome; *HA20 deficiency* haploinsufficiency of A20; *SIFD* sideroblastic anemia with B cell immunodeficiency, periodic fever, and developmental delay

The purpose of this paper is to review the contemporary literature and present an updated list of the imaging findings that may be encountered in patients with an underlying monogenic cause responsible for autoinflammatory disease. All these patients are found to have a genetic mutation that is responsible for their disease. In this paper, we will also investigate the role of imaging in follow-up and present the available abnormal follow up findings that can result either from disease progression or treatment toxicity.

## Materials and methods

This study is a cross-sectional study of a cohort of patients with autoinflammatory diseases who had undergone medical imaging at our institution. Institutional review board approval for this retrospective study was obtained and individual patient consent waived. Patients were selected through clinical and imaging databases. The clinical and imaging findings for patients with disease-causing mutations were reviewed. Gender of the patients, symptoms, and age at first and subsequent presentations, type of mutation, biopsy results when available, and treatment received were recorded. All available medical imaging including radiographs, ultrasound, CT, MRI, nuclear studies, and bone mineral density/DEXA scans were reviewed.

## Results

We found 20 cases of patients with autoinflammatory disease in addition to 14 randomly selected patients with familial Mediterranean fever (FMF). FMF is by far the most common autoinflammatory disease at our institution.

Tables 2, 3, and 4 describe the clinical presentation, underlying gene defect, and imaging findings of the study cohort. Table 1 appendix reports the histopathology results for different biopsies taken from our patient cohort.

**Table 2:** List of the patients of our cohort with summary of the underlying affected gene, and first clinical presentation

Disease	No. of patients	Gender	Gene mutation	Age symptoms started	First clinical presentation
		M:F			
FMF	14^a^	9:5	MEFV gene	Early childhood	Fever, abdominal pain, cervical lymphadenitis, multiple joints pain (hips, knees, ankles, PIPJ)
TRAPS	4	2:2	TNFRSF1A	Childhood	Cervical spine, knees, hips and elbow pain
MKD/HIDS	2	0:2	MVK gene	1 and 5 years	Cervical lymphadenitis, abdominal pain, N/V, arthralgia, myalgia, headache, seizure
NOMID	3	1:2	CAS1 (NLRP3)	2, 6, 9 months	Fever, skin rash, Joint swelling: knees, ankles, elbows, wrists, toes, fingers
MWS	1	0:1	CAS1 (NLRP3)	9 years	Headache
Blau	1	1:0	NOD2	5 months	Rash then swollen joints: knees, ankles, wrists and hands
PAPA	1	1:0	PSTPIP1	5 years	Right knee septic arthritis
DIRA	1	1:0	IL1RN	Newborn	Pustular psoriasis, osteomyelitis
NLRC4-MAS	1	0:1	NLRC4	5 months	Recurrent fever, microcytic anemia, hyperferritinemia, gluten allergy
SIFD	1	0:1	TRNT1	9 weeks	Recurrent fever, profound sensorineural hearing loss and sideroblastic anemia on bone biopsy
HA20 deficiency	2	0:2	TNFAIP3	10 and 15 months	Perianal redness, polyarthritis and vaginal, perineal and mouth ulcers
IL10 def	1	0:1	IL10RB	3 weeks	Abdominal distension and fever
DADA2	1	1:0	CECR1	5 years	Fever, livedo reticularis, stroke, erythema nodosum, medium vessel vasculitis and muscle pain
Aicardi-Goutieres syndrome	1	1:0	SAMHD 1	2 years	Arthritis, nodular rash, chilblains and dysmorphic facies- cold induced reddish papules of the ears, dry red eyes.

*FMF* familial Mediterranean fever; *TRAPS* tumor necrosis factor receptor-associated periodic syndrome; *MKD* Mevalonate kinase deficiency; *HIDS* hyperimmunoglobulinemia D syndrome; *NOMID* neonatal onset multisystem inflammatory disease; *MWS* Muckle-Wells syndrome; *PAPA* pyogenic arthritis, pyoderma gangrenosum and acne; *DIRA* deficiency of interleukin-1 receptor antagonist; *DADA2* deficiency of adenosine deaminase type 2; *IL10 def* interleukin 10 deficiency; *MAS* macrophage activation syndrome; *HA20 deficiency* haploinsufficiency of A20; *SIFD* sideroblastic anemia with B cell immunodeficiency, periodic fever, and developmental delay

^a^Randomly selected patients and not all patient at the hospital

**Table 3:** Imaging findings in the musculoskeletal system, central nervous system, and chest

Disease	Imaging findings		
	MSK	CNS	Chest
FMF	Bilateral sacroiliitis	None	None
TRAPS	None	None	None
MKD/HIDS	None	None	None
NOMID	• Small bilateral knee effusions with synovitis • T6 osteoporotic compression fracture • dysmorphic vertebra	MRI: • Subtle enhancement of vestibular nerves • Macrocephaly • Absent bight posterior pit • Bilateral papilledema • Prominent ventricular and supratentorial CSF spaces • Thin left cerebral convexity subdural collection with pachymeningeal enhancement	None
MWS	None	MRI brain: • Non-specific FLAIR signal in the left centrum Semiovale • Empty sella • Tiny right cerebellar hemisphere focus of bleed or calcification.	None
PAPA	• Growth recovery lines • Right knee: small effusion, extensive synovitis • TMJ: chronic inactive right TMJ arthritis • Right elbow: joint effusion with fluid collection • Hand MRI septic arthritis of the 4th MCPJ with osteomyelitis and adjacent fluid collection at the dorsum of the hand. • Right elbow X-ray: effusion with synovial thickening and enhancement with abscess extending from the joint into the soft tissue anteromedially	None	None
Blau syndrome	• Knee radiographs: effusion and ST swelling around the knees • Ankles and hands, MRI wrists: bilateral tenosynovitis of the extensors and bone edema of the phalangese and metacarpals- improvement after anti-TNF, then worsening, then improvement after Humira and MTX with longitudinal split tear of the extensor carpi-ulnaris, follow up stable minimal synovitis and tenosynovitis- no erosions	None	None
DIRA	• Chest radiographs and CT: chest deformity with multiple bone lesions • Mandible: left lytic lesion • Bone scan: multiple areas of increased uptake, • Hand radiographs: multiple bilateral metaphyseal lytic lesions with cortical thinning and distal phalangeal soft tissue thickening • Heterotopic ossification of the proximal right femur	Brain CT and MRI: posterior circulation parenchymal changes representing ischemia, can be cyclosporin induced (treatment related)	CT chest: • Chronic lung changes (alveolar and interstitial) • Worsening heart disease and pulmonary edema
DADA2	None	MRI brain: • left thalamic infarct- gliosis- recurrent areas of diffusion restriction • Abnormal enhancement of the right intraconal orbital nerve	None
IL10 deficiency	Skeletal survey: delayed maturation-otherwise normal	• US brain at 20 days of life: right parietal subdural collection with meningeal thickening • hematoma on MRI	None
NLRC4-MAS	• Right hip effusion on ultrasound • Delayed skeletal maturation	Brain MRI: focal right cerebellar astrogliosis with widened folia, related to old insult	None
HA20 deficiency	• Osteopenia • Delayed skeletal maturation. • Feet radiographs: 1st MTP joint narrowing, dystrophic calcifications along the tendons of previous injections • Radiographs, CT and MRI of the feet and ankles: bilateral asymmetrical tibiotalar, subtalar and intertarsal arthritis changes with degenerative changes of the right subtalar joint, tenosynovitis and retrocalceneal bursistis	None	None
SIFD	Skeletal survey: growth recovery lines	MRI brain: pachymeningeal enahnecment of porus acousticus	None
Aicardi-Goutieres syndrome	• Spine radiographs: thoracic kyphosis- no scheuermann's disorder • Hands: soft tissue swelling around the right 2, 3, 4 and left 3,4 PIPs. • Delayed skeletal maturation	None	None

*FMF* familial Mediterranean fever; *TRAPS* tumor necrosis factor receptor-associated periodic syndrome; *MKD* mevalonate kinase deficiency; *HIDS* hyperimmunoglobulinemia D syndrome; *NOMID* neonatal onset multisystem inflammatory disease; *MWS* Muckle-Wells syndrome; *PAPA* pyogenic arthritis, pyoderma gangrenosum, and acne; *DIRA* deficiency of interleukin-1 receptor antagonist; *DADA2* deficiency of adenosine deaminase type 2; *IL10 def* interleukin 10 deficiency; *MAS* macrophage activation syndrome; *HA20 deficiency* haploinsufficiency of A20; *SIFD* sideroblastic anemia with B cell immunodeficiency, periodic fever, and developmental delay; *PAN* polyarteritis nodosa; *GI* gastrointestinal; *GU* genitourinary; *MTP* metatarsophalangeal; *CT* computed tomography; *TORCH* toxoplasmosis, other (syphilis, varicella-zoster, parvovirus B19), rubella, cytomegalovirus (CMV), and herpes infections; *TMJ* temporomandibular joint; *MCPJ* metacarpophalangeal joint; *PIP* proximal interphalangeal joint; *MSK* musculoskeletal; *CNS* central nervous system

**Table 4:** Imaging findings in the gastrointestinal system, genitourinary system, lymphadenopathy, and vascular system

Disease	Imaging findings			
	GI	GU	Lymphadenopathy	vascular
FMF	Peritonitis without a primary cause (echogenic fat on ultrasound and fat stranding on CT with small ascites)	None	None	None
TRAPS	Splenomegaly, then resolved	None	None	None
MKD/HIDS	Splenomegaly	None	Bilateral cervical	None
NOMID	None	None	None	None
MWS	None	None	None	None
PAPA	US abdomen: wall thickening of the right colon, splenomegaly	None	None	None
Blau	None	None	None	None
DIRA	None	Renal US: enlarged echogenic kidneys.	None	None
DADA2	None	None	None	Angiogram of the abdominal aorta: suggestive of small vessel arteritis such as PAN
IL10 deficiency	US abdomen: • Marked gallbladder wall thickening • Moderate ascites • Small and large bowel wall thickening with hyperemia and lack of peristalsis, no pneumatosis • Large echogenic liver, • Splenomegaly Lower GI: colitis, terminal ileitis. X-ray: markedly distended bowel loops	Renal ultrasound: • Echogenic kidneys • Abnormal cortical foci, crystal deposition vs. TORCH	None	None
NLRC4-MAS	US abdomen: • Increased periportal echos in the liver • Splenomegaly	None	Mediastinal and intra-abdominal	None
HA20 deficiency	None	None	Axillary and inguinal	None
SIFD	None	None	Submental	None
Aicardi-Goutieres syndrome	None	None	None	None

*FMF* familial Mediterranean fever; *TRAPS* tumor necrosis factor receptor-associated periodic syndrome; *MKD* mevalonate kinase deficiency; *HIDS* hyperimmunoglobulinemia D syndrome; *NOMID* neonatal onset multisystem inflammatory disease; *MWS* Muckle-Wells syndrome; *PAPA* pyogenic arthritis, pyoderma gangrenosum, and acne; *DIRA* deficiency of interleukin-1 receptor antagonist; *DADA2* deficiency of adenosine deaminase type 2; *IL10 def* interleukin 10 deficiency; *MAS* macrophage activation syndrome; *HA20 deficiency* haploinsufficiency of A20; *SIFD* sideroblastic anemia with B cell immunodeficiency, periodic fever, and developmental delay; *PAN* polyarteritis nodosa; *GI* gastrointestinal; *GU* genitourinary; *MTP* metatarsophalangeal; *CT* computed tomography; *TORCH* toxoplasmosis, other (syphilis, varicella-zoster, parvovirus B19), rubella, cytomegalovirus (CMV), and herpes infections; *TMJ* temporomandibular joint; *MCPJ* metacarpophalangeal joint; *PIP* proximal interphalangeal joint; *MSK* musculoskeletal; *CNS* central nervous system

Table 5 lists system-based imaging findings on different imaging modalities previously reported in the English literature for patients with autoinflammatory diseases.

**Table 5:** System-based summary of imaging findings for previously reported patients with autoinflammatory diseases according to different imaging modalities

Disease	Radiographs	Ultrasound	CT	MRI
Musculoskeletal findings				
FMF	• Joint space narrowing • Erosions	Joint effusion		• Myositis • Arthritis • Enthesitis
TRAPS		Joint effusion		• Joint effusion • Myositis • Fasciitis
MKD/HIDS	Periarticular osteopenia			
NOMID	Enlarged, mass like ossified growth plate Widening of the non-ossified part of the physis, cupping and fraying of the metaphysis and resorption of the epiphysis.			Heterogeneous signal with dark calcifications of the enlarged physis on T1- and T2-weighted images with mottled gadolinium enhancement at the physis
MWS	Metaphyseal fraying and cupping and widening of the growth plate			Mottled enhancement of osteochondral junction
Blau	• Non-erosive polyarthritis • Dysplasia	Joint effusion		• Non-erosive polyarthritis • Tenosynovitis
PAPA	• Joint effusion • Joint destruction	Joint effusion		“Similar to septic arthritis” • Joint effusion • Synovitis • Chronic deforming changes
DIRA	• Balloon-like widening of the anterior end of the ribs • Multiple osteolytic lesions • Periosteal elevation along multiple long bones • Recurrent osteomyelitis • Cervical vertebral fusion • Widening of the clavicles • Metaphyseal erosions of the long bones		• Multiple osteolytic lesions • Periosteal cloacking • Heterotopic ossification of the proximal femurs • Recurrent osteomyelitis	
NLRC4-MAS		Joint effusion		Joint effusion
SIFD				
HA20 deficiency	• Asymmetrical non-deforming polyarthritis with involvement of small and large joints			• Synovitis • Tendonitis
IL10 def				
DADA2				
Aicardi-Goutieres syndrome	• Joint contractures • Acro-osteolysis			
CNS findings				
FMF				
TRAPS				
MKD/HIDS			Cerebellar atrophy	Cerebellar atrophy
NOMID				• Meningeal enhancement • Cochlear nerve enhancement
MWS				
Blau			Ischemic stroke	
PAPA			Parenchymal changes related to vasculitis	
DIRA			Parenchymal changes related to vasculitis	
NLRC4-MAS			• Volume loss • Non-specific periventricular white matter signal abnormality	
SIFD				
HA20 deficiency				
IL10 def				
DADA2				
Aicardi-Goutieres syndrome				
Chest findings				
FMF				
TRAPS				
MKD/HIDS				
NOMID				
MWS				
Blau	Interstitial lung disease		Interstitial lung disease	
PAPA				
DIRA				
NLRC4-MAS	Alveolar pulmonary opacities		Alveolar pulmonary opacities	
SIFD				
HA20 deficiency				
IL10 def				
DADA2				
Aicardi-Goutieres syndrome				
Gastrointestinal findings				
FMF	Normal or bowel obstruction due to adhesions	• Peritonitis and small ascites without underlying cause • Bowel obstruction due to adhesions		
TRAPS				
MKD/HIDS				
NOMID				
MWS				
Blau		• Hepatitis (enlarged hypoechoic/heterogeneous liver) • Splenomegaly		
PAPA		• Splenomegaly • Intestinal lesions • IBD similar to Crohn’s disease		
DIRA				
NLRC4-MAS	Air-fluid levels	• Enterocolitis “bowel wall thickening,” small ascites • Hepatosplenomegaly • Gallbladder wall thickening		
SIFD		Hepatosplenomegaly		
HA20 deficiency				
IL10 def		Early onset IBD: • colitis ± small bowel involvement • perianal disease • fistula formation		
DADA2		Hepatosplenomegaly		
Aicardi-Goutieres syndrome				
Genitourinary findings				
FMF				
TRAPS				
MKD/HIDS				
NOMID				
MWS				
Blau		Nephrocalcinosis		
PAPA		Nephrocalcinosis		
DIRA				
NLRC4-MAS				
SIFD		Nephrocalcinosis		
HA20 deficiency				
IL10 def				
DADA2				
Aicardi-Goutieres syndrome				
Lymphadenopathy				
FMF				
TRAPS				
MKD/HIDS				
NOMID				
MWS				
Blau		Generalized lymphadenopathy		
PAPA				
DIRA				
NLRC4-MAS		Mediastinal and abdominal lymphadenopathy		
SIFD				
HA20 deficiency				
IL10 def				
DADA2			Hemorrhagic stroke	
Aicardi- Goutieres syndrome			• Brain calcifications • Leukodystrophy • Cerebral atrophy • Periventricular cysts	
Vascular findings				
FMF				
TRAPS				
MKD/HIDS				
NOMID				
MWS				
Blau			Large vessel vasculitis	
PAPA			CNS vasculitis, aneurysm	
DIRA			CNS vasculitis	
NLRC4-MAS				
SIFD			Multiple enhancing cerebellar lesions (single case)	
HA20 deficiency				
IL10 def				
DADA2			Early onset polyarteritis nodosa (PAN) • Multiple areas of narrowing-beaded appearance of the medium- sized arteries • Micro-aneurysms	
Aicardi-Goutieres syndrome			• Intracranial aneurysm dysplastic vessels	

*FMF* familial Mediterranean fever; *TRAPS* tumor necrosis factor receptor-associated periodic syndrome; *MKD* mevalonate kinase deficiency; *HIDS* hyperimmunoglobulinemia D syndrome; *NOMID* neonatal onset multisystem inflammatory disease; *MWS* Muckle-Wells syndrome; *PAPA* pyogenic arthritis, pyoderma gangrenosum and acne; *DIRA* deficiency of interleukin-1 receptor antagonist; *DADA2* deficiency of adenosine deaminase type 2; *IL10 def* interleukin 10 deficiency; *MAS* macrophage activation syndrome; *HA20 deficiency* haploinsufficiency of A20; *SIFD* sideroblastic anemia with B cell immunodeficiency, periodic fever, and developmental delay; *PAN* polyarteritis nodosa; *MSK* musculoskeletal; *CNS* central nervous system; *IBD* inflammatory bowel disease

## Discussion

## Genetic classification with clinical and imaging findings

### Familial Mediterranean fever

#### Underlying gene defect

Familial Mediterranean fever (FMF) is the most common monogenic autoinflammatory disease with an autosomal recessive inheritance. Mediterranean fever (MEFV) gene mutation encoding pyrin are found in almost all patients with FMF, leading to excess inflammation through increased IL-1β production [3]. It is prevalent among eastern Mediterranean populations, mainly non-Ashkenazi Jews, Armenians, Turks, and Arabs.

#### Clinical information

Clinically, FMF usually has a childhood onset with a mean age of 9.6 ± 8.6 years [4]. It is characterized by recurrent fever attacks which last for 1–3 days and resolve spontaneously associated with polyserositis. In a study of 2838 Turkish FMF patients, the symptoms of patients were fever (92.5%), peritonitis (93.7%), arthritis (47.4%), pleurisy (31.2%), amyloidosis (12.9%), and non-amyloid glomerular disease (0.8%) [4]. The arthritis in FMF patients typically presents as an acute onset of mono- or oligoarthritis predominantly involving the large joints of lower extremities more than those of upper extremities [5, 6]. The arthritis can last for several days leaving no permanent joint damage. Prolonged myalgia, exercise-related leg pain, and sacroiliitis have been reported related to FMF [7]. Myalgia in FMF usually involves a single muscle group featuring pain and enlargement of the affected muscle [8].

In a study of 38 patients with FMF, it was reported that FMF was associated with other diseases in a few patients such as polyarteritis nodosa (in 2 patients), multiple sclerosis (in 1 patient), and autoimmune hemolytic anemia (in 1 patient) [6]. In a small cohort of 33 patients with inflammatory bowel disease, it has been found the rate of disease-causing *MEFV* mutations and FMF disease is higher in patients with Crohn’s disease compared to the rest of the Turkish population [9]. Over the years, it has been found that FMF can be associated with Henoch-Schonlein purpura, polyarteritis nodosa, and seronegative spondyloarthropathy and these are more common among patients with FMF compared to the rest of the normal population [4].

#### Imaging findings

Abdominal imaging is frequently performed in patients with FMF due to acute abdominal pain and is usually normal but might show findings related to peritonitis in the form of diffuse intra-abdominal echogenic fat on ultrasound and fat stranding, mesenteric venous congestion, and small ascites on CT scan, without an underlying cause such as appendicitis evident. Recurrent peritonitis may cause adhesions leading to intestinal obstruction.

MRI can demonstrate myositis showing focal ill-defined areas of increased signal intensity within the clinically painful musculature on T2-weighted images [8]. MRI is also ideal to reveal joint effusion and soft tissue edema in acute onset arthritis. In the work of Eshed et al., 91% of the 11 patients with FMF had enthesitis of the Achilles tendon, long plantar ligament, or the plantar fascia, including enthesophytes, erosions, and bone marrow edema; 80% of the 11 patients had radiographic signs of sacroiliitis [5].

**Fig. 2:**
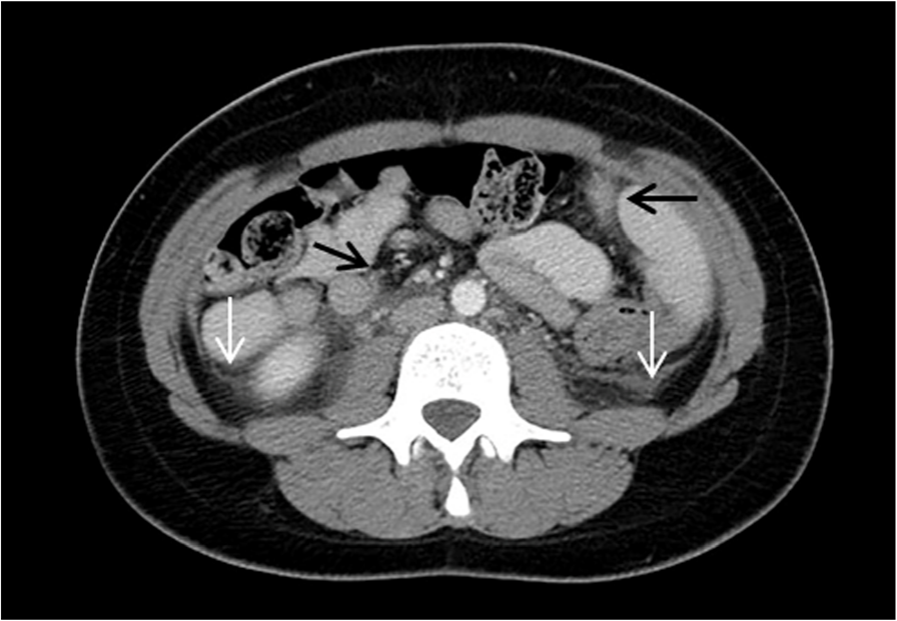
Familial Mediterranean fever. Selected axial CT image of the abdomen after oral and intravenous contrast administration in a 14-year-old girl presenting to the Emergency department with acute abdominal pain showing small ascites (white arrows) and diffuse intra-abdominal fat stranding (black arrows). Solid organs, bowel, and appendix were normal (not shown). Findings represent peritonitis without detectable underlying cause

**Fig. 3:**
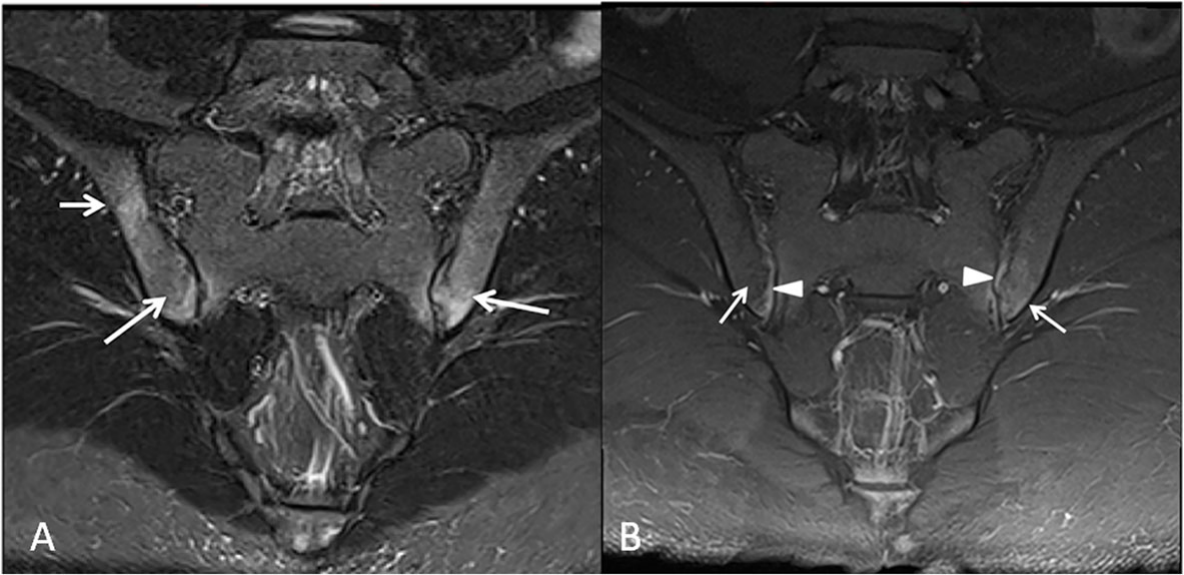
A 17-year-old girl with Familial Mediterranean Fever. **a** Coronal oblique Short-Tau inversion recovery (STIR) of the sacroiliac joints demonstrates abnormal subarticular bone marrow signal (arrows). **b** Coronal T1-post intravenous gadolinium administration demonstrates bone marrow (arrows) and synovial (arrow heads) enhancement. Findings are consistent with sacroiliitis

In our study, 14 patients with FMF were randomly selected and reviewed. Most of them presented with fever and acute abdomen and their abdominal imaging findings were normal. One of the patients showed evidence of peritonitis on US and CT scan without a secondary cause (Fig. 2). Another 17-year-old patient was incidentally found to have sacroiliitis on MR-enterography performed for recurrent abdominal pain and fever (Fig. 3).

### Tumor necrosis factor receptor-associated periodic syndrome

#### Underlying gene defect

Tumor necrosis factor receptor-associated periodic syndrome (TRAPS) is the second most common monogenic autoinflammatory disorder and the most common autosomal dominant monogenic autoinflammatory disorder [10]. It results from mutations in the tumor necrosis factor receptor superfamily member 1A (TNFRSF1A) gene (12p13) encoding the 55-kD receptor for tumor necrosis factor-α (TNF-α) [10]. This leads to the development of symptoms related to unopposed action of tumor necrosis factor (TNF).

#### Clinical information

The disease onset is in childhood and early adulthood. It is characterized by recurrent episodes of long-lasting (usually1–3 weeks) fever, migratory rash, and migratory pain that start proximally then migrate distally. Painful conjunctivitis and periorbital swelling are distinguishing features of TRAPS [11]. Less specific but frequent manifestations are fatigue and symptoms of inflammation in different regions of the body, such as the musculoskeletal system, gastrointestinal tract, and serosal membranes. Abdominal pain is a consistent feature which can be related to peritonitis or inflammation of the abdominal wall muscles [12, 13]. Inflammation of the bowel wall with ulceration has been reported in the literature on endoscopic evaluation [12, 13]. Arthralgia and myalgia are common. Chest pain, headaches, and testicular pain may also occur [14]. Two-thirds of patients will have arthralgia during the attacks, demonstrating monoarticular or oligoarticular distribution, mainly affecting large joints such as knees, hips, and shoulders. However, small joints like fingers and temporomandibular joints may also be affected [14]. Myalgia may be present during the attacks, usually involving a single muscle group at the root of a limb. Attacks can be triggered by stress, minor infection, or vigorous exercise.

### Imaging findings

Ultrasound or MRI may reveal joint effusion in the affected joint(s). MRI can also demonstrate associated myositis or fasciitis [14–16]. MRI in Dode’s work showed a segmental inflammatory process in the affected muscle and subcutaneous tissue without affecting adjacent joints or fascia [15]. In a biopsy-proven monocytic fasciitis in an adult case associated with TRAPS, the MRI demonstrated edematous changes in the left quadriceps compartment and surrounding soft tissues including fascia and dermis [16]. However, no muscle abnormality was evident on pathology. The author argues that TRAPS myalgia is caused by fasciitis rather by myositis. CT and MRI of the abdomen may show circumferential wall thickening of the small bowel with surrounding inflammatory changes and lymphadenopathy [12].

In our study, four patients presented with disease-causing mutation; two of them (50%) demonstrating polyarticular complaints but normal radiographs.

### Mevalonate kinase deficiency

#### Underlying gene defect

Mevalonate kinase deficiency (MKD) is an autosomal recessive disease caused by loss-of-function mutations in the mevalonate kinase (MVK) gene. The MVK gene encodes for mevalonate kinase which is a critical enzyme involved in cholesterol biosynthesis [17].

#### Clinical information

MKD has a wide spectrum of phenotypes with variable disease severity depending on the remaining MVK activity. The two well-established phenotypes at the ends of the spectrum are mevalonic aciduria (MA) which is the severe fatal form and hyperimmunoglobulinemia D syndrome (HIDS) which is the mild form [18]. Patients with MA present during the neonatal period with periodic fever, severe neurological impairment, cerebellar ataxia, growth retardation, and death [18]. HIDS usually presents with an early childhood onset, mostly within the first year of life, but can manifest during the first 5 years of life [11]. The main clinical features are recurrent fever, rash, painful cervical lymphadenopathy, headache, mouth ulcers, and gastrointestinal symptoms. More than 60% of the patients may have arthralgia with or without arthritis, mainly involving large joints such as the knee [11].

#### Imaging findings

Cerebellar atrophy has been reported in patients with mevalonic aciduria [11, 19]. Radiographic findings of the affected joints in patients with HIDS are usually normal or demonstrate soft tissue edema/swelling and/or periarticular osteopenia. Neither joint destruction nor erosions have been reported in the literature.

### Cryopyrin-associated periodic syndromes

Cryopyrin-associated periodic syndromes (CAPS) is a group of autoinflammatory disorders that contains three forms or variants. The most severe form is the neonatal onset multisystem inflammatory disease (NOMID), also known as chronic infantile neurologic cutaneous and articular (CINCA) syndrome. A less severe form is the Muckle-Wells syndrome and the mildest form is the familial cold-induced autoinflammatory syndrome (FCAS). Patients with CAPS present with periodic fever and urticarial rash. Other features may differ and contribute to the severity of the disease. Patients with FCAS present with cold-induced fever, neutrophilic urticaria, and inflammatory hearing loss. FCAS will not be further discussed in this paper as there are no reported imaging findings in our cohort nor in the literature.

#### Underlying gene defect

CAPS are usually associated with missense mutations in the *NLRP3* gene, which lead to interleukin-1β overproduction. Clinical response to IL-1 receptor blocker proves this mechanism of the disease. CAPS are inherited disorders caused by an autosomal dominant mutation, although sporadic cases have been reported, especially in NOMID [20].

#### Neonatal-onset multisystem inflammatory disease

#### Clinical information

Neonatal-onset multisystem inflammatory disease (NOMID) is a severe neonatal or early infancy monogenic autoinflammatory disease. NOMID is characterized clinically by fever, chronic urticarial rash, arthropathy, and central nervous system (CNS) manifestations including chronic aseptic meningitis, hydrocephalus, severe headache, papilledema, and hearing loss. Severe bone overgrowth abnormalities may develop in the extremities. Approximately 60% of NOMID patients have prominent arthropathy, most commonly involving the knees [21]. Patients with NOMID are usually short in stature with valgus or varus deformity of the knees. These abnormalities begin in infancy and cause changes that persist beyond skeletal maturity, leaving the patient with leg length discrepancy and skeletal deformity that result in early degenerative arthropathy and joint contractures [21, 22].

#### Imaging findings

The reason behind the osseous manifestations in NOMID is the abnormal endochondral bone growth associated with this syndrome which leads to bony overgrowth of the physis [22, 23]. The areas commonly affected are around the knees and around the ankles. Radiographically, the disease starts with widening of the non-ossified part of the physis followed by cupping and fraying of the metaphysis and may cause resorption of the epiphysis [23]. The hallmark of the disease is enlarged, mass-like ossified growth plate [21, 23]. The distinctive enlarged and deformed areas develop in patients with NOMID without evidence of synovitis [21]. MRI may demonstrate heterogeneous signal with dark calcifications of the enlarged physis on T1- and T2-weighted images with mottled gadolinium enhancement at the physis [23].

Patients may show features of meningitis on brain MRI with leptomeningeal and pachymeningeal thickening and variable enhancement. Cochlear nerve enhancement has been reported as well. It has been reported that the degree of meningeal and cochlear nerve enhancement decreases after treatment with anakinra, the IL-1 receptor blocker [24].

**Fig. 4:**
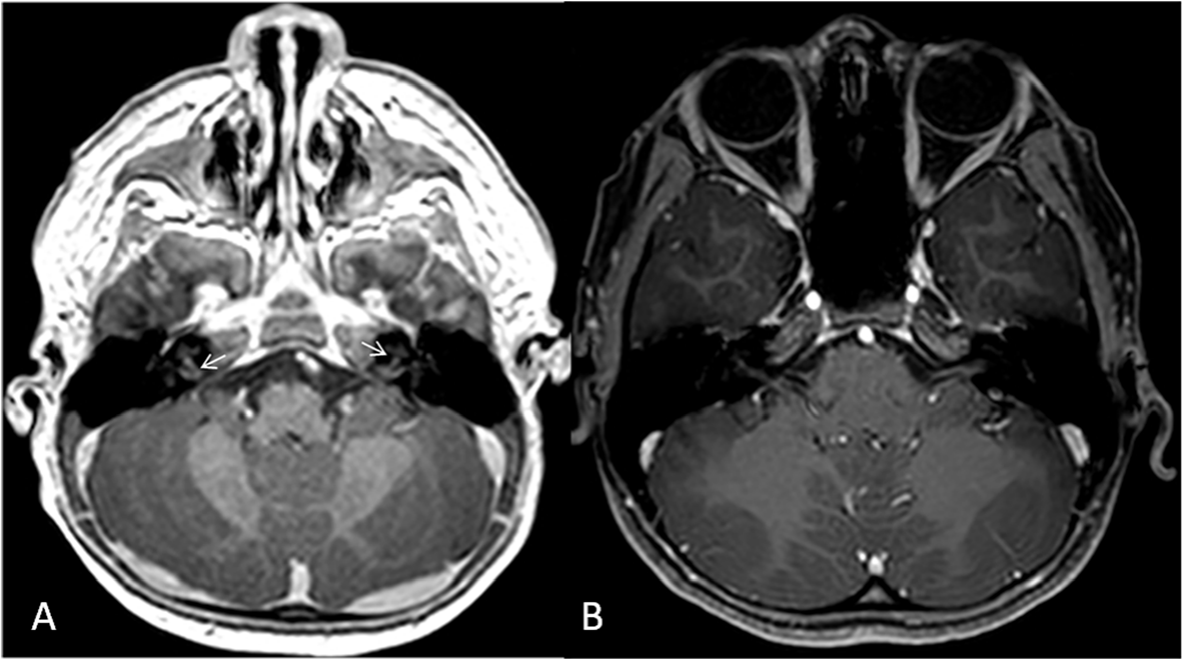
A 1-year-old girl newly diagnosed with neonatal onset multisystem inflammatory disease (NOMID), first presenting with recurrent fever and urticarial rash. MRI of the brain including internal auditory canals was performed as part of the assessment. **a** Selected axial 3D T1- post intravenous gadolinium administration demonstrates subtle enhancement of the cochlear nerves bilaterally (arrows). **b** Enhancement resolved in 6 years follow up after treatment with anakinra

Our patients did not show the classic osseous imaging findings. Early diagnosis and treatment of these patients may have prevented the development of previously reported osseous deformities. One of our patients showed subtle enhancement of cochlear nerve on brain MRI that resolved after treatment with anakinra (Fig. 4).

### Muckle-Wells syndrome

#### Clinical information

Muckle-Wells syndrome (MWS) is an autosomal dominant disease in most of the cases and characterized by recurrent urticaria-like skin rashes, hypoacusia, conjunctivitis, and amyloidosis [25]. Patients present with urticarial rash, low-grade fever, and arthralgia. Recurrent arthritis and conjunctivitis episodes frequently occur. During severe attacks, patients often complain of headache and aseptic meningitis, and some MWS patients present with papilledema. Sensorineural hearing loss is the most discriminatory clinical feature from FCAS and is due to chronic inflammation in the inner ear, likely leading to damage of the Corti organ [26].

#### Imaging findings

Radiographs of the painful joint are usually negative, or may reveal non-destructive polyarthritis or oligoarthritis affecting large joints [12, 27].

Multiple sclerosis-like brain lesions in MRI has been reported in a 45-year-old female patient [28].

Our study patient with disease-causing mutation, *CAS1* (*NLRP3*), presented with headache at the age of 9 years. Her brain MRI showed non-specific findings which do not explain her symptom (Table 3).

### Blau syndrome—also known as pediatric granulomatous arthritis

#### Underlying gene defect

Blau syndrome is a rare autosomal dominant monogenic autoinflammatory disease caused by mutations in the nucleotide-binding oligomerization domain-containing protein 2 [*NOD2*] gene, also called *CARD15* [29, 30]. *NOD2* mutation leads to early-onset recurrent inflammation of the skin, synovium, and uveal tract. Blau syndrome was previously believed to represent early-onset sarcoidosis due to the clinical similarities and as both diseases share the same mutations on *NOD2* in 50–90% of the cases [31]. However, Rybicki et al. found that they do not share the same locus [32].

#### Clinical information

The prevalence of the disease is unavailable in the literature [31, 33]. Patients with Blau syndrome present symptoms of the disease before 4 years of age [31]. Median onset for articular disease is 2 years (range, 3 months–13 years). Median disease duration is 12.8 years (range, 1.1–57 years) [30]. Typical clinical findings include a triad of fine maculopapular skin rash, severe often treatment-resistant uveitis and granulomatous arthritis with exuberant tenosynovitis. Noncaseating granulomas are typically seen in synovial biopsy of the affected joints.

The most common and earliest presenting symptom is skin rash. MSK manifestations include polyarticular synovitis and tenosynovitis. Additional systemic visceral involvement has also been reported, including hepatic and renal granulomas, large- to medium-vessel vasculitis and CNS involvement [29–31].

#### Imaging findings

Arthritis is the most common manifestation of the disease, being present in more than 90% of the reported cases [30, 34]. It is described as “boggy arthritis” and commonly is painless, non-erosive, and presents with polyarticular rather than oligoarticular involvement. It can be symmetric in up to 40% of cases. Although non-specific, almost all previously published radiographs for patients with Blau arthritis showed periarticular osteopenia which is caused by the increase of blood flow to the inflamed joints and surrounding soft tissue.

In Ikeda’s study, ultrasound was used to assess the swollen joints in ten patients with Blau syndrome [35]. These authors reported a high proportion of involved joints presenting with tenosynovitis rather than intra-articular synovitis (41.5% versus 27.9%). Superficial surface erosions can be seen in several proximal interphalangeal (PIP) joints of hands and mild joint space narrowing can be present in large joints (Fig. 6). Camptodactyly, flexion contracture of the fingers and toes due to arthritis and tenosynovitis, is one of the features of the disease.

**Fig. 5:**
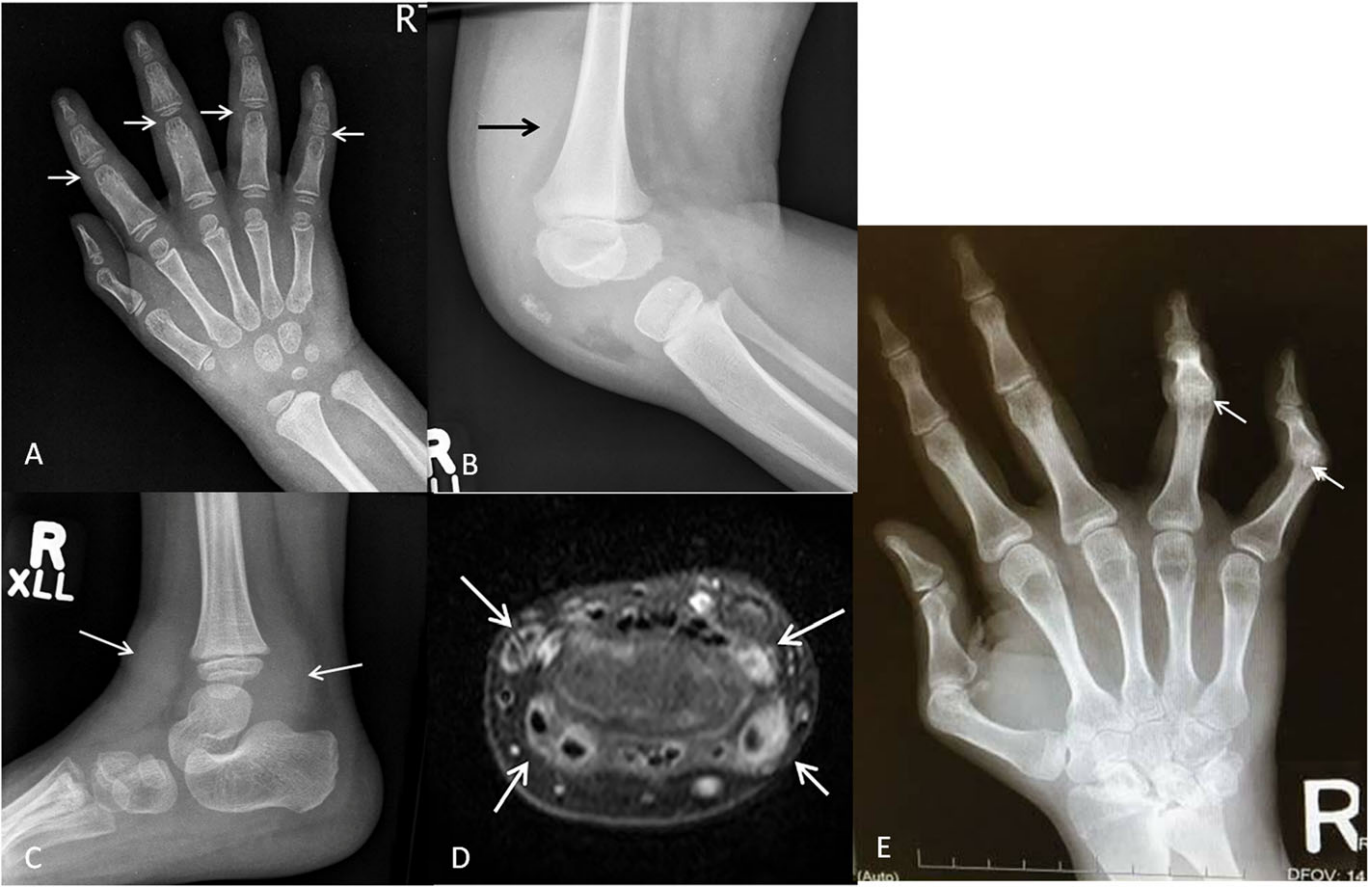
A 2-year-old girl with Blau syndrome presenting with swelling of the hands, knees, and ankles. **a** Frontal radiograph of the right hand demonstrates diffuse periarticular osteopenia, soft tissue swelling around the proximal interphalangeal joints (arrows). No joint space narrowing, erosions or osseous destruction. Similar changes were present in the left hand (not shown). **b** Lateral radiograph of the right knee demonstrates large suprapatellar joint effusion (arrow) and soft tissue swelling. Similar changes were seen in the left knee (not shown). **c** Lateral radiograph of the right ankle demonstrates soft tissue swelling along the tendons representing tenosynovitis (arrows). **d** Axial T1-weighted image with fat suppression after intravenous contrast administration of the right wrist demonstrate thickening and enhancement of the tendon sheaths of all the extensor tendons (arrows) representing tenosynovitis. **e** follow up frontal radiograph of the right hand of the same patient at the age of 25 years demonstrates typical flexion contracture of the PIP joints of the fourth and fifth digits (arrows). Note that there are no erosions

In Rosé et al.’s study, the radiographs of 31 patients with Blau syndrome showed involvement of the wrists (87%) of cases, knees (73%), ankles (63%), and PIP joints (53%) [30]. One-third of patients had involvement of metacarpophalangeal joints and/or elbows. Rarely involved joints at onset were hips (9%), spine (6%), and temporomandibular joints (3%). Dysplastic osseous changes were seen in two-thirds of the patients which included flexion contracture limited to the proximal interphalangeal joints (Fig. 5), carpal dysplasia with carpal crowding mainly the proximal row, an abnormal distal radial epiphysis with biconcave articular surface, an abnormal shape with a shortened distal ulna, and long and thin diaphysis of the second metacarpal bone [30].

Visceral and vascular manifestations were reported in 52% of the patients in the study by Rosé et al. [30]. These manifestations included granulomatous hepatitis, splenomegaly, generalized lymphadenopathy, interstitial lung disease (not described further in the paper), nephrocalcinosis, transient facial palsy, ischemic stroke, and large-vessel vasculitis. Recurrent fever and erythema nodosum were reported as expanded clinical manifestations of the disease which were never reported previously.

The CT imaging findings of the only reported case of Blau syndrome with proposed interstitial lung disease (pneumonitis) were described as several small ground-glass opacities involving the medial segment of the right middle lobe and lower lung lobes. Scattered enlarged axillary and mediastinal lymph nodes were seen [36]. However, no lung biopsy was performed. Biopsy of an enlarged retroauricular lymph node of that patient showed noncaseating granuloma and granulomatous lymphadenitis.

Our patient with Blau disease first presented with macular rash followed by multiple joint swelling. Radiographs showed knee effusions and ankle soft tissue swelling in a distribution presumed to be related to tenosynovitis. MRI of the hands showed tenosynovitis (Fig. 5).

### Pyogenic arthritis, pyoderma gangrenosum, and acne syndrome

#### Underlying gene defect

Pyogenic arthritis, pyoderma gangrenosum, and acne (PAPA) syndrome is a rare autosomal dominant autoinflammatory syndrome caused by missense mutations in the *PSTPIP1* gene located in chromosome 15. The exact mechanism by which the mutant gene causes the syndrome is not fully understood but it leads to overproduction of IL-1β in certain cells [37].

#### Clinical information

The disease is very rare as only 53 cases have been reported in the literature up to the best of our knowledge [38]. Despite its name, the classic clinical triad of pyogenic arthritis, pyoderma gangrenosum, and acne is rarely observed [38, 39]. Patients with PAPA syndrome typically present with pyogenic sterile arthritis that recurs in childhood and is often triggered by minor trauma. This tends to improve by adolescence, while the skin manifestations, pyoderma gangrenosum, and severe cystic acne exacerbate instead. However, clinical presentation may vary and include recurrent fever, arthralgia, and aseptic arthritis. Cultures from skin and joints are sterile [37–39].

#### Imaging findings

Joint effusion with synovial vegetations has been reported and indicates synovitis associated with the clinical diagnosis of arthritis [38]. Advanced degenerative arthritis has been reported in one elbow, both hips, and both knees of a patient with PAPA syndrome [40]. Significant joint destruction with resultant physical disability and impaired quality of life can develop [26, 38]. It is difficult to distinguish PAPA joint involvement from infection based on images only and septic arthritis should be clinically excluded.

A diaphyseal osteolytic lesion was previously described in a single case and is not a characteristic feature in PAPA but can be seen commonly in Deficiency of interleukin 1 receptor antagonist (DIRA) and CRMO [41].

Besides the triad of symptoms, other organ involvement is rare with a few case reports describing visceral and vascular involvement in patients with the syndrome. Splenomegaly, nephrocalcinosis, and intestinal lesions have been reported in a single patient with genetically proven PAPA syndrome [37]. The intestinal lesions were described as multiple colon ulcers similar to those of Crohn’s disease with ileocecal stenosis and perianal disease; however, histopathology showed no granuloma. Cerebrovascular findings in the form of CNS vasculitis have been reported in one patient in the English literature [42]. This patient developed subcortical white matter signal changes on brain MRI during disease flare. CTA at that time showed vascular wall irregularity of the anterior and posterior circulations. Later on, the patient developed an aneurysm of the right posterior cerebral artery complicated by rupture that was treated with endovascular coiling [42].

**Fig. 6:**
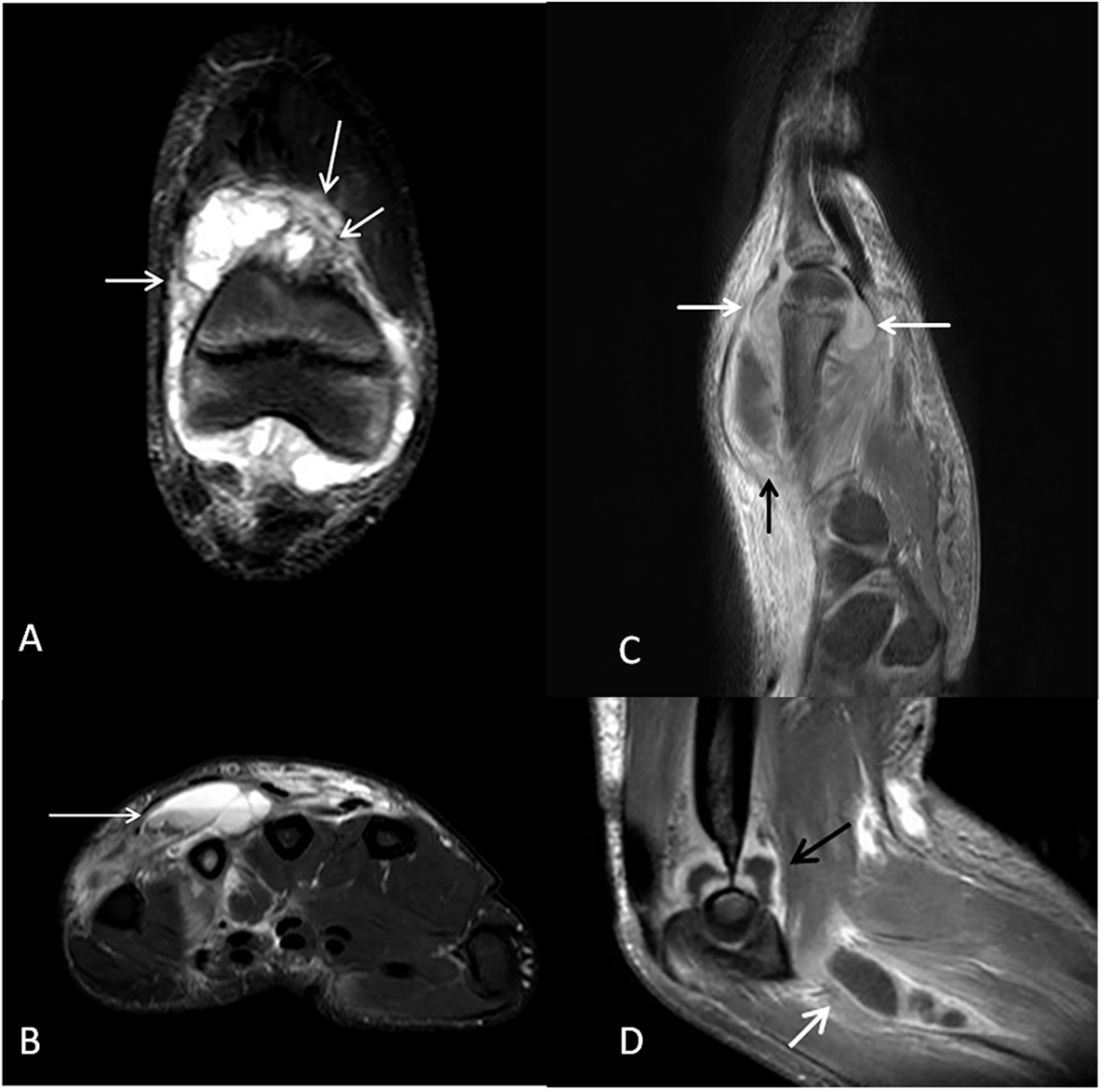
Different presentations of osteoarticular inflammatory involvement in a girl with pyogenic sterile arthritis, pyoderma gangrenosum and acne (PAPA) syndrome. **a** Coronal STIR of the anterior right knee demonstrates moderate effusion with synovial thickening (arrows) and surrounding soft tissue edema. **b** Axial STIR image at the mid-level of the metacarpals of the left hand demonstrates abnormal bone marrow signal of the fourth metacarpal bone with an associated loculated dorsal fluid collection that demonstrates a fluid-fluid level (arrow). **c** Sagittal T1-weighted image with fat suppression and intravenous contrast administration through the fourth metacarpal bone at the same presentation demonstrates ring enhancement of the dorsal fluid collection (black arrow). Moreover, there is extensive synovial thickening and enhancement of the fourth metacarpophalangeal joint (white arrows). **d** Sagittal T1-weigthed image of the right elbow with fat suppression and after intravenous gadolinium administration demonstrates moderate effusion with synovial thickening and enhancement (black arrow). There is a ring-enhancing fluid collection (abscess) extending from the joint into the soft tissue antero-medially (white arrow)

Our study patient with PAPA syndrome started at the age of 5 years to have recurrent episodes of arthralgia, joint swelling, morning stiffness, and reduced range of motion with occasional urticarial rash around the involved joints. The joint involvement was mono- or oligo-articular with or without previous eliciting trauma. The patient presented each episode with similar or different joint involvement compared to the previous episode and the involved joints included the knees, left ankle, right elbow, right temporomandibular joint, and the fourth metacarpophalangeal joint of the right hand. Organisms were never isolated from the joint aspirate thus representing sterile pyogenic arthritis. MRI of the involved joints showed extensive synovitis and joint effusions, fluid collections, and chronic deforming joint changes (Fig. 6).

### Deficiency of interleukin-1 receptor antagonist

#### Underlying gene defect

DIRA results from homozygous mutation or deletion in the gene (*IL1RN*) that leads to secretion of truncated protein (interleukin-1 receptor antagonist) that has no control on the regulation of the interleukin inflammatory pathway [43].

#### Clinical information

The hallmark of this disease is osseous and skin involvement in the form of osteomyelitis, periosteitis, and pustulosis.

In Aksentijevich et al.’s study which reported 9 patients with DIRA, all patients presented with fetal distress, pustular rash, joint swelling, oral mucosal lesions, and pain with movement [43]. Over time, these patients developed cutaneous pustulosis ranging from discrete crops of pustules to generalized severe pustulosis or ichthyosiform lesions. Other less common manifestations were hepatosplenomegaly, CNS vasculitis, interstitial lung disease, and failure to thrive. Interestingly, none of the patients had fever, but all had marked elevation of the erythrocyte sedimentation rate and C-reactive protein. Bone biopsies were performed in two patients which were sterile. Histopathology showed purulent osteomyelitis with fibrosis and sclerosis.

#### Imaging findings

The most common findings in patients with DIRA are balloon-like widening of the anterior end of the ribs, periosteal elevation along multiple long bones, multiple sterile osteolytic lesions, periosteal cloaking, and heterotopic ossification of the proximal femurs [43]. Other less common findings are cervical vertebral fusion, widening of the clavicles, and metaphyseal erosions of the long bones. Regarding CNS vasculitis, the MRI findings of one reported patient showed infarction of the right post-central gyrus in the acute phase and encephalomacia on follow up imaging [43].

**Fig. 7:**
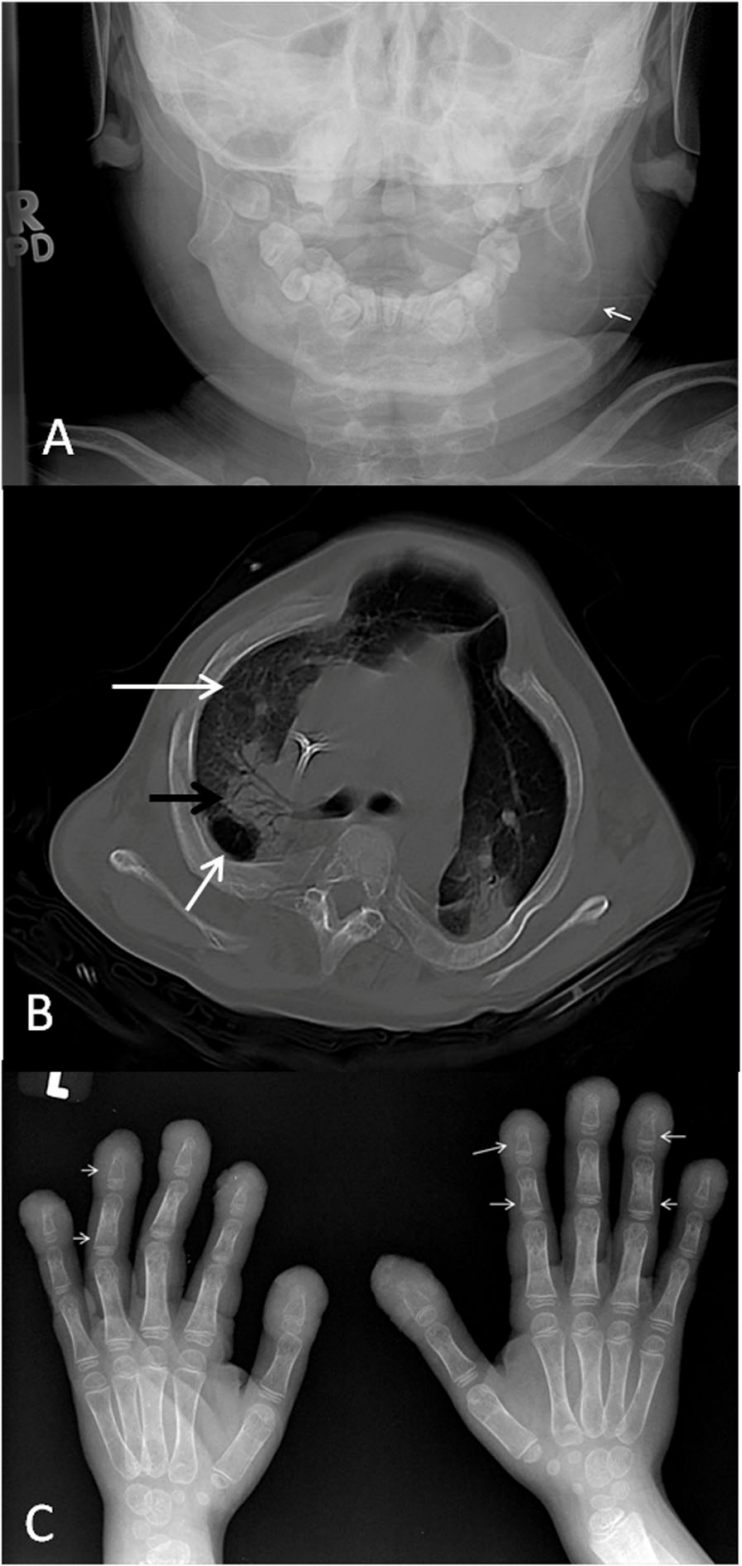
Several imaging findings in a male patient with deficiency of interleukin 1 receptor antagonist (DIRA). **a** Posteroanterior view of the mandible at the age of 7 years when he presented with left mandibular swelling shows a well-defined lytic lesion within the body of the left mandible with expansion of the bone and thinning of the cortex (arrow). There are no aggressive features. **b** Axial unenhanced CT of the chest in bone window demonstrates diffuse broadening of the ribs with ground glass density of the medulla and thinning of the cortex. Bone biopsy showed fibrous changes indicative of healing that is likely related to the patient’s bone disease. There are alveolar (black arrow) and interstitial (long white arrow) pulmonary opacities and nodules with cyst formation (short white arrow). Lung biopsy: pulmonary hemosiderosis. **c** Anteroposterior radiograph of the hands performed when the patient presented with abnormal nails demonstrates multiple bilateral symmetrical metaphyseal lytic lesions with associated cortical thinning (arrows). There is distal phalangeal soft tissue thickening

Our study patient with DIRA presented as a newborn with pustular psoriasis and subsequent presentations with recurrent osteomyelitis and proximal right femoral heterotopic ossification. Images of Fig. 7 demonstrate different types of osseous involvement.

### *NLRC4*-related macrophage activation syndrome

#### Underlying genetic defect

*NLRC4* gain-of-function gene mutations lead to activation of the NLRC4 inflammasome that leads to oversecretion of proinflammatory molecules including IL-1β and, even more prominently, IL-18 [44].

#### Clinical information

Patients with NLRC4 mutations present with early-onset recurrent fever flares, recurrent macrophage associated syndrome (MAS), and/or enterocolitis [44]. NLRC4 is highly expressed in intestinal epithelial cells and that explains the intestinal manifestation in patients with mutations in this gene. Cold-induced or spontaneous episodic fever and urticaria, and CNS inflammation are less common manifestations of the disease.

#### Imaging findings

There are no specific features of this disease. To our knowledge, imaging findings related to enterocolitis have never been reported. However, endoscopic evaluation for a recently published case with a proven disease-causing mutation showed severe mucosal ulcerations and inflammation extending from her stomach to the large bowel [45].

Reported findings related to MAS are hepatomegaly, splenomegaly, gallbladder wall thickening, alveolar pulmonary opacities, and volume loss of the brain with non-specific periventricular white matter signal abnormality [44]. If left untreated, MAS can progress in some cases to coagulopathy, multiorgan failure, acute respiratory distress syndrome (ARDS), and death.

Our study patient with proven disease-causing mutation presented at 5 years of age with history of recurrent fever, microcytic anemia, hyperferritinemia, and gluten allergy. Over the disease course, she manifested features of right hip effusion, mediastinal and abdominal lymphadenopathy, splenomegaly, and focal right cerebellar astrogliosis which could be related to a previous insult. She ultimately responded well to anakinra.

### Sideroblastic anemia with B cell immunodeficiency, periodic fever, and developmental delay

#### Underlying gene defect

Sideroblastic anemia with B cell immunodeficiency, periodic fever, and developmental delay (SIFD) is caused by biallelic loss of function mutation in *TRNT1* gene leading to a defect in cytoplasmic and mitochondrial tRNA [46].

#### Clinical information

Apparently, only 18 cases with SIFD have been reported so far [46, 47]. This disorder has features of autoinflammation and autoimmunity; however, patients with SIFD have variable onset ranging from neonatal period to adulthood with variable presentation ranging from inflammatory episodes to isolated retinitis pigmentosa [47]. As the name indicates, patients usually have sideroblastic anemia with immunodeficiency, periodic fever, and developmental delay. Other less common non-specific reported features are diarrhea, vomiting, sensory neural deafness, seizure, ataxia, retinitis pigmentosia, brittle hair, hepatosplenomegaly, and nephrocalcinosis [47].

#### Imaging findings

There is no pathognomic imaging feature for SIFD. One of the reported patients with seizure had an MRI before passing away that showed multiple enhancing cerebellar lesions. However, the histopathology of these lesions in this case was not reported [47].

### Haploinsufficiency of A20

#### Underlying gene defect

Haploinsufficiency of A20 (HA20) develops in patients who have heterozygous loss-of-function mutations in *TNFAIP3* gene encoding the A20 protein. Decreased expression of this protein leads to activation of NF-κB, an important inflammatory pathway, leading to increased inflammation [48].

#### Clinical information

Although the clinical features are highly variable even in the same family and with the same mutation, the hallmark of HA20 is early-onset recurrent painful oral and genital ulcers with or without gastrointestinal ulcers [49]. Other reported manifestations are gastrointestinal (such as abdominal pain, bloody diarrhea due to intestinal inflammation, and perforation) and MSK manifestations (including arthralgia and polyarthritis) as well as episodic fever, and recurrent infections. Cutaneous, ocular, and cardiac manifestations are less common. Lupus nephritis and CNS vasculitis were reported in only one patient so far, to our knowledge [49]. Most of the reported cases have developed symptoms during infancy or childhood; however, a few developed symptoms in their 20s [49]. Because of the similarity of the symptoms of patients with HA20 and patients with symptoms of patients with Behcet disease, almost 70% of the patients with HA20 were misdiagnosed with Behcet disease before the definitive diagnosis was made.

#### Imaging findings

Radiographs of patients with HA20 deficiency may present with asymmetrical non-deforming polyarthritis, involvement of small and large joints, and tendonitis [49].

**Fig. 8:**
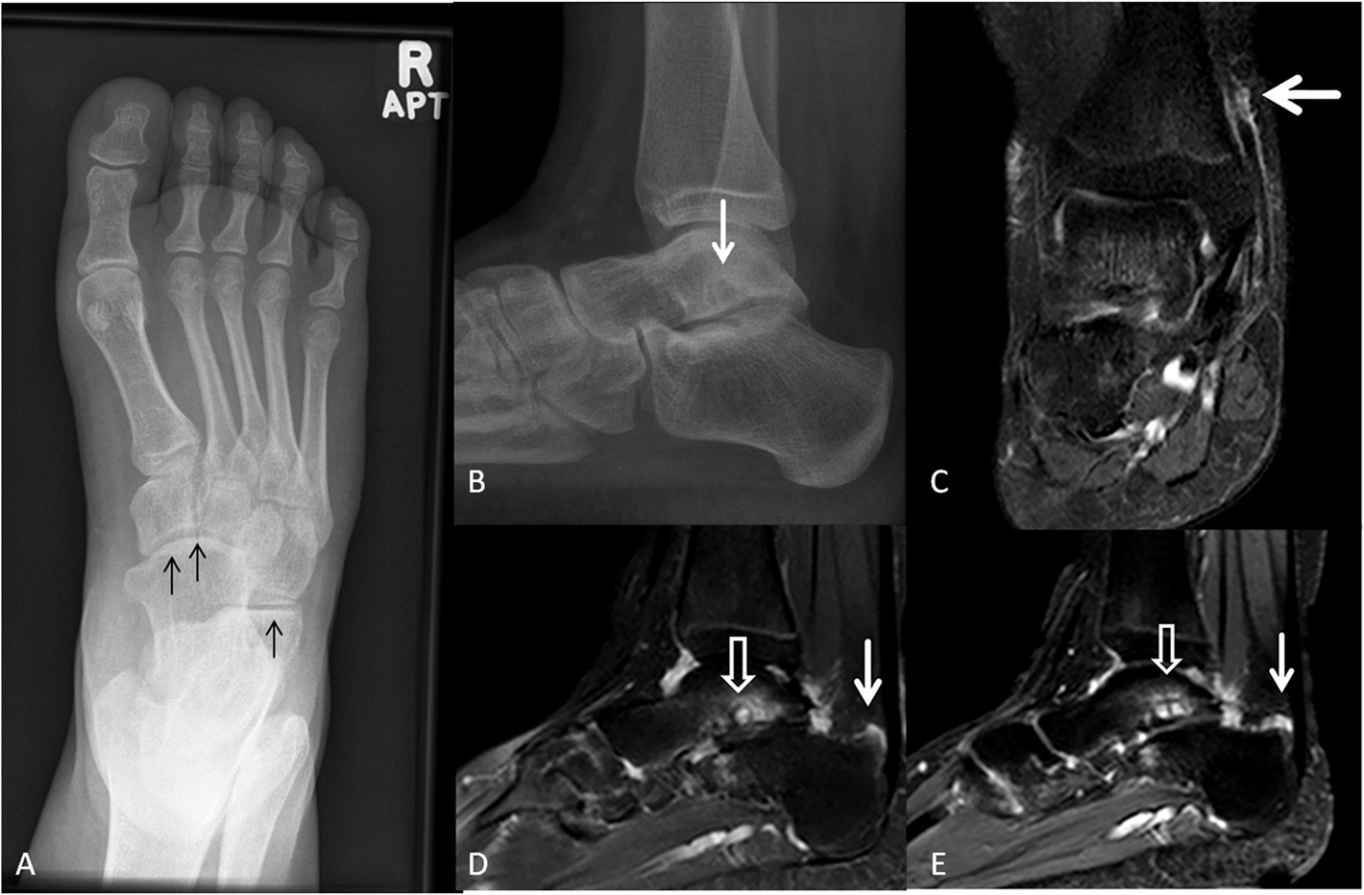
A 14-year-old girl with HA20 presented with pain in her ankles and feet. **a** Anteroposterior radiograph of the right foot demonstrates joint space narrowing of the talonavicular and calcaneocuboid joints (arrows). Similar but asymmetrical changes are present in the left foot (not shown). **b** Lateral radiograph of the right ankle demonstrates subtalar joint space narrowing with subchondral sclerosis and subarticular cystic changes of the inferior surface of the talus (arrow). **c** Coronal STIR of the right ankle demonstrates fluid tracking along the tibialis posterior tendon (arrow) with post gadolinium enhancement (not shown). **d** Sagittal STIR and **e** T1-weighted image with fat suppression and intravenous gadolinium administration of the right ankle demonstrate subtalar joint space narrowing and subchondral cystic changes with surrounding edema involving the inferior articular surface of the talus and contrast enhancement (empty white arrow). There is joint space narrowing and small effusion in the tibiotalar, subtalar, and talonavicular joints with synovial contrast enhancement. There is retrocalceneal bursistis (white arrow). Arthritis, tenosynovitis, and retrocalceneal bursistis are likely inflammatory with degenerative changes of the subtalar joint

One of our two patients with HA20 deficiency presented with polyarthritis of the feet, most severe disease in the right foot (Fig. 8). The other patient had no abnormal imaging findings.

### IL-10 deficiency

#### Underlying gene defect

Loss of function mutations affecting any of the two genes encoding interleukin-10 receptor (*IL10RA or IL10RB* genes); or affecting the gene encoding the cytokine IL-10 (*IL*-*10* gene), will cause early-onset inflammatory bowel disease. This stimulates TNF production and hence proinflammatory cytokines overproduction [48].

#### Clinical information

Patients with IL-10 deficiency present with remitting and relapsing symptoms of inflammatory bowel disease (IBD) at a younger age compared to patients with polygenic IBD disease where symptoms peak within the second decade of life. These symptoms include bloody or non-bloody diarrhea, abdominal pain, fever, and perianal disease. Patients with early-onset IBD manifested during their first year of life are usually more prone to have recurrent disease compared to patients with later disease onset whom can have intractable course [50]. Early-onset IBD can present as Crohn’s disease, ulcerative colitis (UC), or unclassified IBD [50]. Complicated perianal disease and enteric fistulae have been reported [51, 52].

#### Imaging findings

**Fig. 9:**
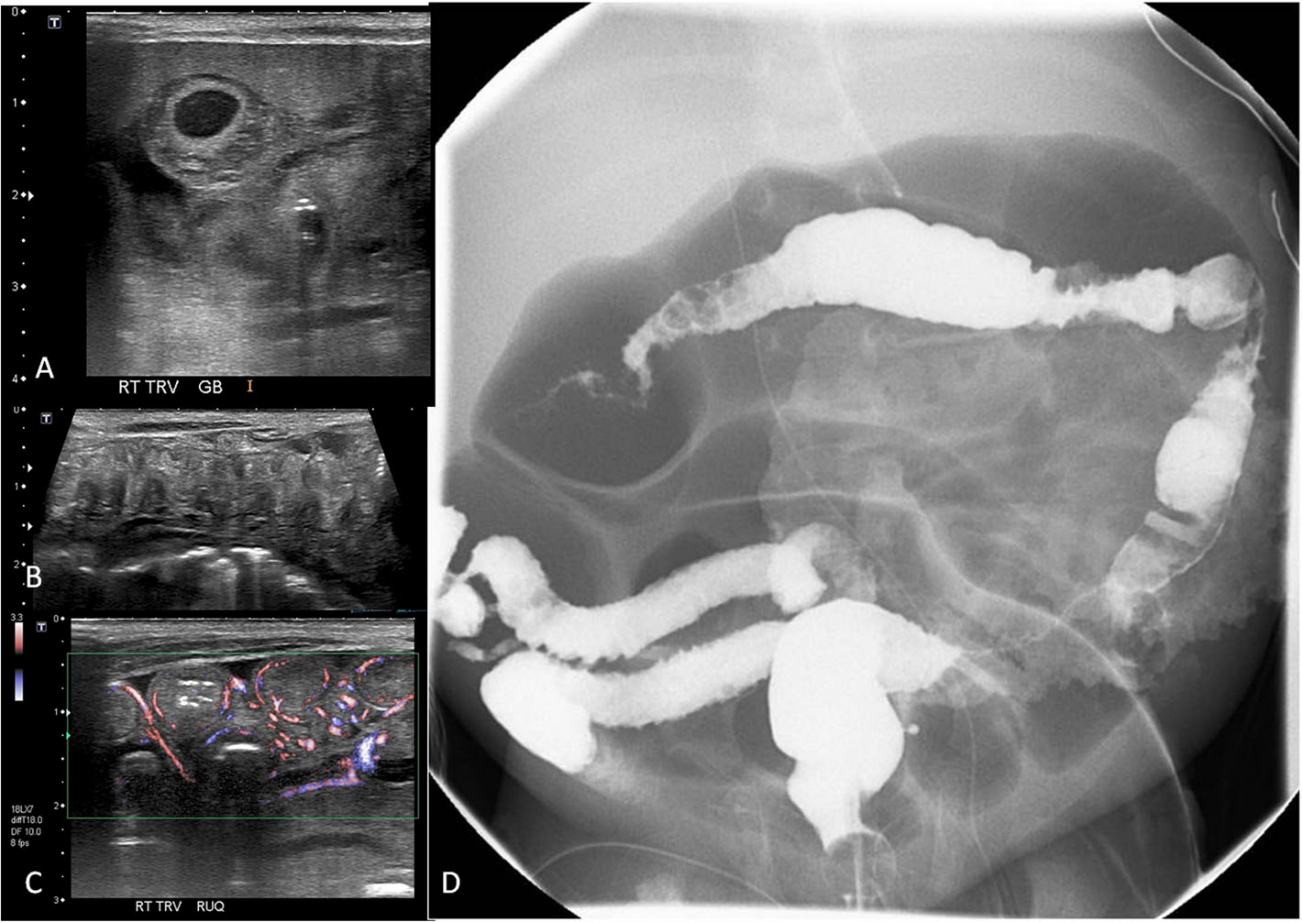
A 5-week-old baby girl with bilious vomiting and abdominal distension. **a**–**c** Selected images of abdominal ultrasound demonstrate **a** marked gallbladder wall thickening and (**b**) small bowel wall thickening and hyperemia and (**c**) transverse colon wall thickening. There was lack of peristalsis on real time ultrasound and moderate ascites (not shown). There was no pneumatosis intestinalis. **d** Frontal fluoroscopic view of contrast enema performed at 8 weeks of life due to persistent abdominal distension and bloody diarrhea: the contrast is filling the colon, terminal ileum, and appendix: there is mildly reduced caliber of the colon with multiple areas of wall irregularities of the colon and terminal ileum. Note the massively dilated small bowel loops at the center of the abdomen. This patient is found to have IL-10 deficiency and positive genetic testing (IL10RB)

These patients have classic imaging findings of Crohn’s and UC, including colitis with or without small bowel involvement (Fig. 9), perianal disease, rectovaginal, and anovaginal fistulae.

### Deficiency of adenosine deaminase type 2

#### Underlying gene defect

This disease is caused by loss-of-function mutations in the *CECR1* (now known as *DAD2*) gene, which encodes adenosine deaminase 2 protein. Mutations were detected in 17/48 (35.4%) patients in a prior multicenter study [53]. It is inherited in autosomal recessive pattern. In cases with strong clinical suspicion for this disease but negative genetic testing, functional assay of the enzyme activity (ADA2) should help confirm the diagnosis.

#### Clinical presentation

Patients with the reported gene mutation present with clinical symptoms between 6 months and 7 years, while those with no detectable gene mutation present with symptoms later in life [53]. Patients with ADA2 deficiency classically present with early onset polyarteritis nodosa (PAN) in addition to hemorrhagic and ischemic stroke. Recurrent fever, hepatosplenomegaly, livedo reticularis, immunodeficiency, and anemia/cytopenia are also features of the disease.

#### Imaging findings

Neuroimaging typically confirms the clinical diagnosis of stroke. The reported infarctions in these patients can involve the subcortical white matter and deep gray matter [53, 54]. Features of PAN in this disease include areas of infarction of the affected part of the organ and pruning and irregularity of the medium and small arteries in angiogram with development of microaneurysms in the distal branches.

**Fig. 10:**
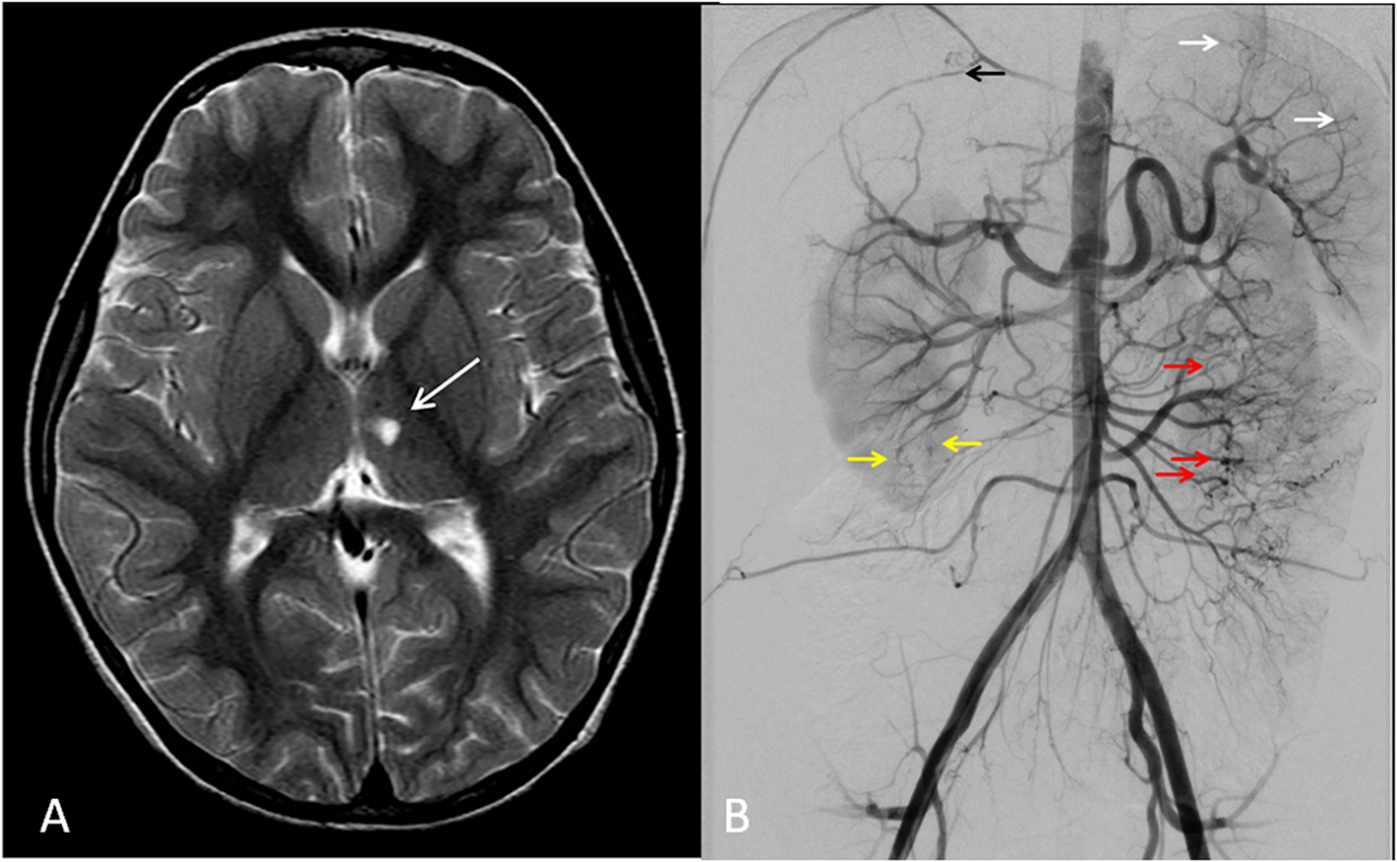
A 12-year-old male with deficiency of adenosine deaminase type 2 (DADA2) and long history of recurrent fever, left thalamic stroke at 5 years of age, livedo reticularis, and erythema nodosum: **a** Axial T2-weighted image demonstrates high signal left thalamic lesion with mild negative mass effect (arrow), no surrounding edema and no diffusion restriction (not shown), indicative of old infarct. **b** Aortic angiogram through a right femoral puncture access: pruning, distal obliteration and irregularity of the intra-hepatic arteries (black arrow). There are multiple microaneurysms in the distal branches of the splenic artery (white arrows) and intra renal arteries of the right kidney (yellow arrows). There is hypervascularity of the jejunum with multiple irregularities and micro-aneurysms involving the jejunal branches (red arrows). Findings are in keeping with small vessel vasculitis such as polyarteritis nodosa (PAN). This patient tested positive for disease-causing gene mutation (CECR1)

Our study patient presented with recurrent fever, history of left thalamic stroke, livedo reticularis, and with radiologic and histological features of polyarteritis nodosa (PAN) on abdominal angiogram and skin biopsy, respectively, at 5 years of age (Fig. 10).

### Aicardi-Goutieres Syndrome

#### Underlying gene defect

Aicardi-Goutieres syndrome (AGS) is an early-onset encephalopathy that can be caused by a gene mutation in one of 7 discovered genes so far (TREX1, RNASEH2B, RNASEH2C, RNASEH2A, SAMHD1, ADAR1, and IFIH1) [55]. This leads to an increase in serum and cerebrospinal fluid (CSF) alpha-interferon, CSF leukocytosis, and calcifying cerebral microangiopathy [56].

#### Clinical presentation

Clinical presentation and age at onset may vary related to the mutated gene. AGS caused by mutations in TREX1, RNASEH2A, and RNASEH2C usually presents early in the neonatal period as “pseudo-TORCH” manifestations with severe neurological dysfunction in the form of progressive microcephaly, spasticity, psychomotor retardation, and may lead to death [57]. Less severe disease is commonly caused by mutations in RNASEH2B, SAMHD1, and ADAR1 which present later in childhood with less intense neurological manifestations [57]. Skin and neurological manifestations are seen in most of the patients such as chilblain lesions, livedo reticularis, cortical blindness, poor head control, and trunk hypotonia. However, not all patients with disease-causing mutations have neurological involvement [57]. Other less common manifestations are arthritis, myositis, thrombocytopenia, and occasional sterile fever [58].

#### Imaging findings

Brain calcifications, leukodystrophy, and cerebral atrophy are the classic hallmark of the disease and are seen in most of the patients. However, the distribution of the calcifications and the pattern of white matter involvement with or without other imaging findings, such as periventricular cysts and vascular lesions, may vary according to the mutated gene [55]. Deep white matter cyst formation is more commonly seen with TREX1 mutations while vascular lesions such as aneurysm and dysplastic vessels are seen in 50% of the patients with SAMHED1 gene mutation, which present later in life [55, 57].

Joint contractures, caused by chilblain lesions and acro-osteolysis, have been reported in some phenotypes [59].

**Fig. 11:**
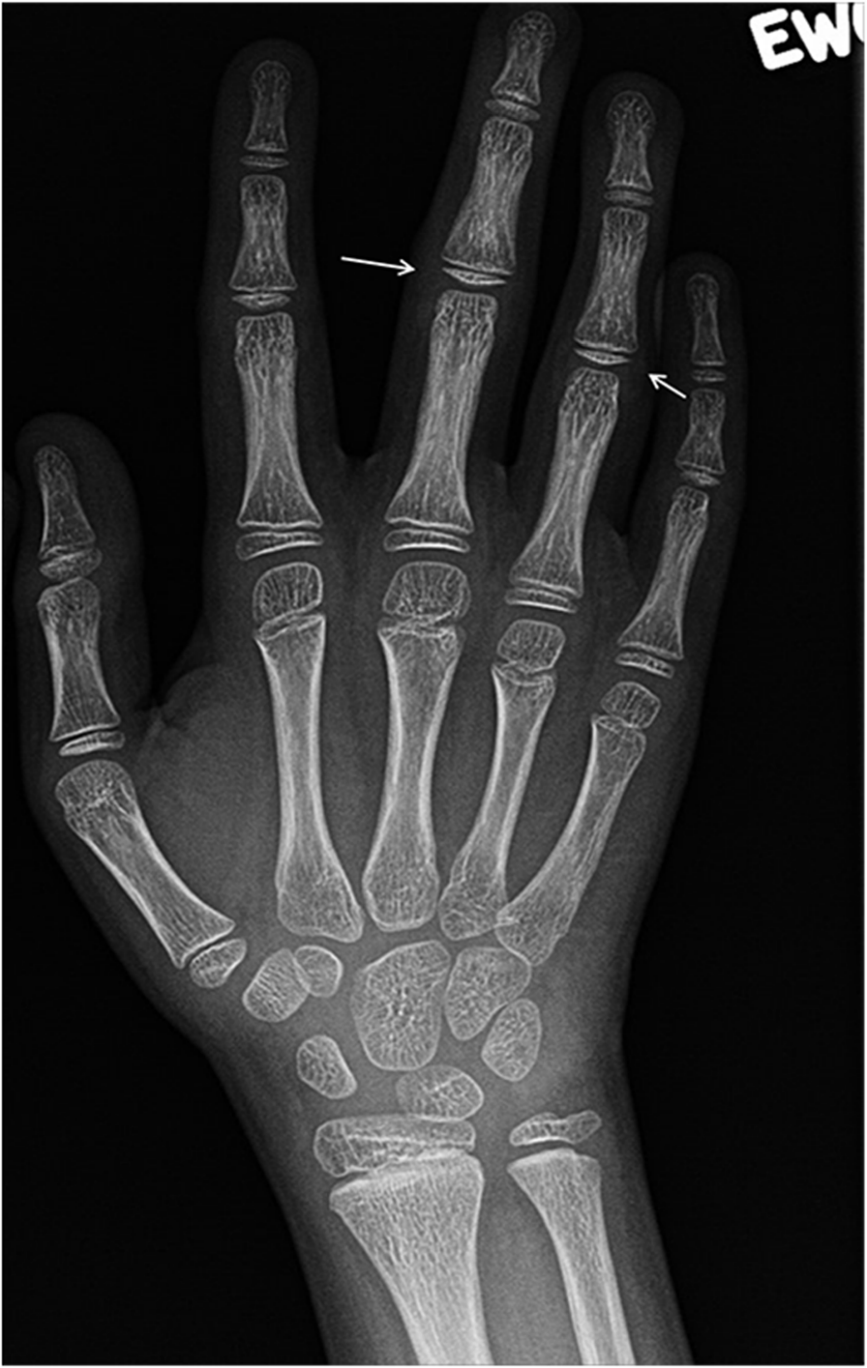
A 13-year-old girl with Aicardi-Goutieres syndrome. She has long history of arthritis, nodular rash, and chilblains started at the age of 2 years. Anteroposterior radiograph of the right hand demonstrates diffuse periarticular osteopenia, soft tissue swelling of the proximal interphalangeal joints (PIPs), mainly the third and fourth PIPs (arrows). There are no erosions, joint space narrowing or soft tissue calcifications. Similar changes are present in the left hand (not shown)

Radiographic findings of arthritis in our patient with AGS were peri-articular osteopenia and soft tissue thickening around the involved joints of his hands and feet without bone erosions (Fig. 11).

### Follow up imaging and autoinflammatory disease damage index

**Fig. 12:**
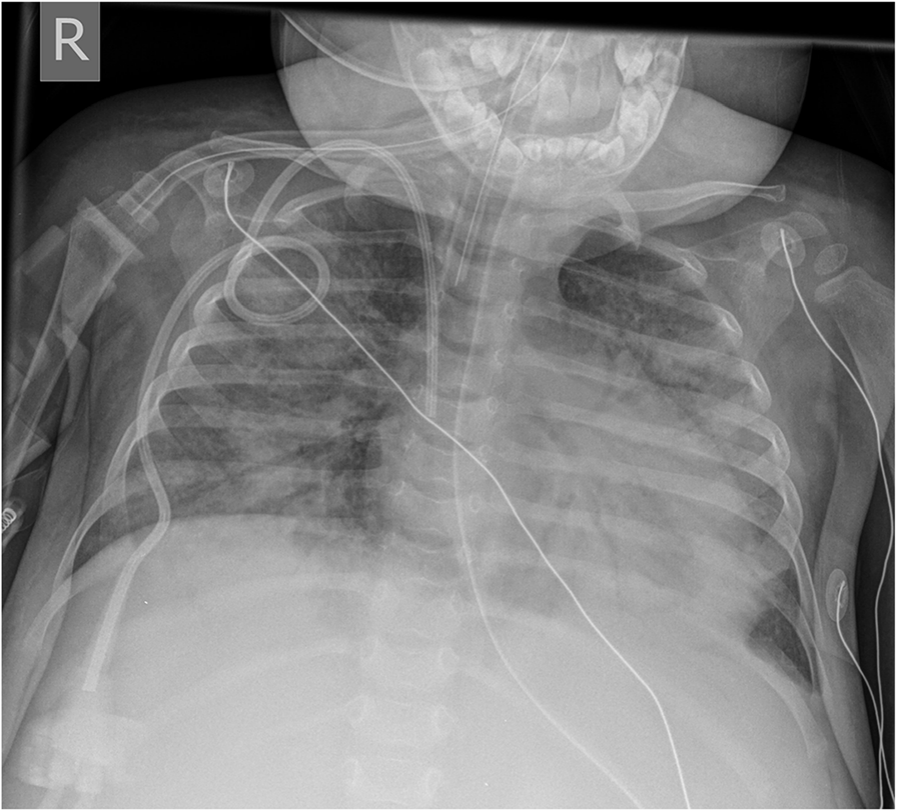
Supine portable chest radiograph for a 15-month-old girl with sideroblastic anemia and immunodeficiency, periodic fever and developmental delay (SIFD), 1 month after bone marrow transplant: the radiograph demonstrates diffuse bilateral pulmonary opacities without cardiomegaly. Note the endotracheal tube and the right central venous line. The patient died with acute respiratory distress syndrome due to graft-versus-host disease

After establishment of the diagnosis, clinical and laboratory follow up are crucial to ensure good control of the disease. The clinical picture and inflammatory markers are checked during clinical visits. Medical imaging can be used as one of the tools to assess the disease burden on first presentation and on follow up to assess treatment response or treatment toxicity in term of organ damage and persistent loss of structure or function of the organ. Our patient with NOMID is an example of treatment response (anakinra) with resolved bilateral cochlear nerve enhancement (Fig. 4). Figure 12 is an example of treatment-related complication in a patient with SIFD after bone marrow transplantation.

Whole body MRI is rarely used up to the best of our knowledge neither in the initial assessment nor in the follow up of patients with monogenic autoinflammatory diseases, unless they have features that are suggestive of chronic non-bacterial osteomyelitis (CNO), in which case they are managed according to recommendations for CNO. These disorders include DIRA and Majeed syndrome. The rarity of these diseases may play a role in that. Another reason is that patients usually present with single system/organ involvement that is best assessed by dedicated imaging.

In this study, we focused on primary imaging diagnosis of the aforementioned diseases due to the fact that the authors of this study do not have a schedule for follow-up imaging in this group of diseases in clinical practice, consonant to the routine protocols for assessment of these diseases in other pediatric healthcare centres around the world. Table 6 shows preferential imaging used for primary diagnostic assessment of different monogenic autoinflammatory diseases described in this study.

**Table 6:** General advice concerning algorithms for primary imaging of patients with autoinflammatory diseases

Disease	Presenting symptom/clinical question	Imaging modality
FMF	Abdominal pain	- Abdominal radiograph and US - If negative aforementioned examinations and ongoing clinical concern: CT (oral and intravenous contrast) or MRI. Injection of gadolinium for MRI will depend on pre-contrast enhancement findings. - In case of chromic enteritis: MRE
	Joint pain	- Initial imaging: X-rays - Ultrasound: if suspicion of fluid or synovitis - MRI (contrast-enhanced): synovitis and osteochondral changes
TRAPS	Myositis/fasciitis	MRI (contrast-enhanced if suspicion of superimposed collection or osteomyelitis)
MKD/HIDS	Cerebellar ataxia	MRI (contrast-enhanced if suspicion of cerebellitis)
	Joint pain	- Initial imaging: X-rays - Ultrasound: if suspicion of joint fluid or synovitis MRI (contrast-enhanced): synovitis and osteochondral changes
NOMID	CNS/hearing manifestations	MRI of brain and inner ear (contrast-enhanced)
	Bone and joint abnormalities	- Initial imaging: X-rays - Ultrasound: if suspicion of fluid or synovitis MRI (contrast-enhanced): for assessment of synovitis and osteochondral changes
MWS	Joint pain	- Initial imaging: X-rays - Ultrasound: if suspicion of fluid or synovitis MRI (contrast-enhanced): for assessment of synovitis and osteochondral changes
	Headache, CNS manifestations	MRI of brain (inner ear) (contrast-enhanced)
Blau	Joint manifestations	- Initial imaging: X-rays - Ultrasound: if suspicion of fluid or synovitis MRI (contrast-enhanced): for assessment of synovitis and osteochondral changes
	Hepatic and renal granulomas	Ultrasound of abdomen (including high-resolution scans)
	Renal medullary nephrocalcinosis	Ultrasound of kidneys (including high-resolution scans)
	Interstitial lung disease	CT of the chest (high resolution)
	Stroke	MRI of brain (contrast-enhanced)
	Lymphadenitis/swelling	Ultrasound (gray-scale) of region-of-interest (including color/power Doppler)
	Large vessel vasculitis	US, MRA or CTA (in rare circumstances conventional angiogram
PAPA	Arthritis	- Initial imaging: X-rays - Ultrasound: if suspicion of fluid or synovitis MRI (contrast-enhanced): for assessment of synovitis and osteochondral changes
	Nephrocalcinosis	Ultrasound of kidneys (including high-resolution scans)
	Intestinal lesions	Ultrasound (gray-scale and color/power Dopler), MRE
	Perianal disease	MRI of the pelvis (with small field-of-view and use of gadolinium)
	CNS vasculitis	MRI of the brain including MRA or CTA
DIRA	MSK manifestations	- Initial imaging: X-rays - Ultrasound: if suspicion of fluid or synovitis MRI (contrast-enhanced): for assessment of synovitis and osteochondral changes
	CNS vasculitis	MRI of the brain (contrast-enhanced)
NLRC4-MAS	Enterocolitis	Ultrasound of the bowel (with color/power Doppler)
SIFD	Hepatosplenomegaly and Nephrocalcinosis	Ultrasound of abdomen
HA20 deficiency	Joint pain/arthritis	- Initial imaging: X-rays - Ultrasound: if suspicion of fluid or synovitis MRI (contrast-enhanced): for assessment of synovitis and osteochondral changes
IL10 def	Early onset inflammatory bowel disease	Ultrasound then MRE
	Perianal disease	MRI of the pelvis (with small field-of-view and use of gadolinium)
DADA2	Stroke	MRI of the brain with MRA
	Vasculitis	US, MRA or CTA. Rarely, conventional angiogram
Aicardi-Goutieres syndrome	CNS manifestations	Brain CT and MRI (depending on the diagnosis, use of gadolinium).
	MSK manifestations	- Initial imaging: X-rays - Ultrasound: if suspicion of fluid or synovitis MRI (contrast-enhanced): for assessment of synovitis and osteochondral changes

*FMF* familial Mediterranean fever; *TRAPS* tumor necrosis factor receptor-associated periodic syndrome; *MKD* Mevalonate kinase deficiency; *HIDS* hyperimmunoglobulinemia D syndrome; *NOMID* neonatal onset multisystem inflammatory disease; *MWS* Muckle-Wells syndrome; *PAPA* pyogenic arthritis, pyoderma gangrenosum, and acne; *DIRA* Deficiency of interleukin-1 receptor antagonist; *DADA2* deficiency of adenosine deaminase type 2; *IL10 def* interleukin 10 deficiency; *MAS* macrophage activation syndrome; *HA20 deficiency* haploinsufficiency of A20; *SIFD* sideroblastic anemia with B cell immunodeficiency, periodic fever, and developmental delay; *CNS* central nervous system; *US* ultrasound; *CT* computed tomography; *MRE* MR enterography; *MRA* magnetic resonance angiogram; *CTA* computed tomography angiogram; *MSK* musculoskeletal

The autoinflammatory disease damage index was developed to assess end organ damage in the four most common hereditary monogenetic diseases: FMF, TRAPS, CAPS, and HIDS [1]. However, the main reason this index was developed is to analyze the outcome of the patient groups [1]. Joint restriction (due to destructive arthritis and joint contractures) and osseous deformity are examples of musculoskeletal involvement in patients with NOMID due to disease progression. It is important to mention that patients may develop these manifestations on their first presentation due to late diagnosis or may have progressed after diagnosis due to treatment unavailability/inaccessibility. Steroids are a commonly used medication in the management of patients with autoinflammatory diseases and have a known list of complications including obesity, hypertension, osteoporosis, cataract, and failure to thrive. A systematic approach in dealing with these patients is important as any organ can be affected by the disease itself or the medications. CNS, ocular, ear, serosal, hepatic, renal/amyloidosis, and gynecological involvement are reported. Psychological health can be affected in the form of depression [1].

The index is clinically based and the role of imaging in this is directed to answer the clinical question when new symptoms develop or to follow up the previously detected lesions/changes. Knowledge of the potential complications is important to provide better service to these patients with these rare diseases.

## Conclusion

Autoinflammatory diseases are relatively rare entities that can affect any system of the body. Given many nonspecific imaging features for most diseases within the spectrum of abnormalities, awareness of findings of these diseases and good communication with clinicians may aid in reaching an accurate diagnosis and allow earlier more targeted therapies.

## Data Availability

Not applicable.

## Abbreviations

ADDI: Autoinflammatory disease damage index
AGS: Aicardi-Goutieres syndrome
ARDS: Acute respiratory distress syndrome
CAPS: Cryopyrin-associated periodic syndromes
CINCA syndrome: Chronic infantile neurologic cutaneous and articular syndrome
CNS: Central nervous system
CRMO: Chronic recurrent multifocal osteomyelitis
CSF: Cerebrospinal fluid
DADA2: Deficiency of adenosine deaminase type 2
DIRA: Deficiency of interleukin 1 receptor antagonist
FCAS: Familial cold-induced autoinflammatory syndrome
FMF: Familial Mediterranean fever
HA20: Haploinsufficiency of A20
HIDS: Hyperimmunoglobulinemia D syndrome
IBD: Inflammatory bowel disease
IL-1: Interleukin1
MA: Mevalonic aciduria
MAS: Macrophage activation syndrome
MKD: Mevalonate kinase deficiency
MWS: Muckle-Wells syndrome
NOMID: Neonatal onset multisystem inflammatory disease
PAN: Polyarteritis nodosa
PAPA syndrome: Pyogenic arthritis, pyoderma gangrenosum and acne syndrome
PIP joints: Proximal interphalangeal joints
SIFD: Sideroblastic anemia with B cell immunodeficiency, periodic fever, and developmental delay
TNF-α: Tumor necrosis factor-α
TNFRSF1A: Tumor necrosis factor receptor superfamily member 1A
TORCH: Toxoplasmosis, Others (like syphilis), Rubella, Cytomegalovirus, Herpes infections
TRAPS: Tumor necrosis factor receptor-associated periodic syndrome
UC: Ulcerative colitis
